# Systematic pan-cancer analysis of tumour purity

**DOI:** 10.1038/ncomms9971

**Published:** 2015-12-04

**Authors:** Dvir Aran, Marina Sirota, Atul J. Butte

**Affiliations:** 1 Institute for Computational Health Sciences, University of California, San Francisco, California 94158, USA; 2 Division of System Medicine, Department of Pediatrics, Stanford University, Stanford, California 94305, USA

## Abstract

The tumour microenvironment is the non-cancerous cells present in and around a tumour, including mainly immune cells, but also fibroblasts and cells that comprise supporting blood vessels. These non-cancerous components of the tumour may play an important role in cancer biology. They also have a strong influence on the genomic analysis of tumour samples, and may alter the biological interpretation of results. Here we present a systematic analysis using different measurement modalities of tumour purity in >10,000 samples across 21 cancer types from the Cancer Genome Atlas. Patients are stratified according to clinical features in an attempt to detect clinical differences driven by purity levels. We demonstrate the confounding effect of tumour purity on correlating and clustering tumours with transcriptomics data. Finally, using a differential expression method that accounts for tumour purity, we find an immunotherapy gene signature in several cancer types that is not detected by traditional differential expression analyses.

## Abstract (web summary)

The importance of the tumour microenvironment has now been realised, however the presence of non-tumour cells in cancer samples can complicate genomic analyses. Here, the authors estimate tumour purity in 10,000 samples from the TCGA dataset and can detect a signature of T cell activation.

## Graphical abstract

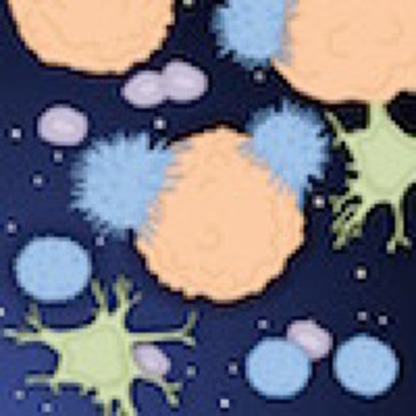

The tumour microenvironment is a complex milieu consisting of factors that promote growth and inhibit it, as well as nutrients, chemokines, and very importantly, other non-cancerous cell types. These cells include fibroblasts, immune cells, endothelial cells and normal epithelial cells^1^. All of these constituents interact with one another and with the tumour as it grows. This admixture is thought to have an important role in tumour growth, disease progression and drug resistance^2^,^3^. Notably, infiltrating immune cells, and particularly infiltrating T lymphocytes, have been associated with tumour growth, invasion and metastasis in several cancer types^4^,^5^.

Tumour purity is the proportion of cancer cells in the admixture. Until recently, it was estimated by a pathologist, primarily by visual or image analysis of tumour cells. With the advancement of genomic technologies, many new computational methods have arisen to infer tumour purity. These methods make estimates using different types of genomic information, such as gene expression^6^, somatic copy-number variation^7^,^8^,^9^ somatic mutations^7^,^10^ and DNA methylation^7^,^11^. Estimates made by these methods are generally consistent with one another, though, to date, no systematic sensitivity analysis in multiple cancer types has been performed.

The Cancer Genome Atlas (TCGA) is currently the largest available data set for genomic analysis of tumours. It contains over 10,000 pretreatment samples across 30 cancer types and includes measurements such as RNA sequencing (RNA-seq), DNA methylation, copy-number variation and more^12^. The consortium had originally set a quality threshold that tumour samples included in the cohort be composed of at least 80% tumour nuclei, as determined by visual analysis^13^. However, this threshold was later reduced to 60%. Given the status of TCGA as a flagship project of the National Cancer Institute, we assumed that sample purity was the best possible using current conventional sample acquisition methods, and we thus hypothesized that differences in purity were due more to properties of the cancers, and less to the acquisition method. While TCGA argues that 60% purity is sufficient to distinguish the tumour’s signal from those of other cells, it remains to be evaluated if this level of purity across tumour samples affects the interpretation of genomic analyses.

In recent years, sporadic analyses have sought to determine tumour purity levels and take them into account during analysis^14^,^15^,^16^,^17^,^18^,^19^,^20^,^21^. These studies used different purity estimation methods and tested only specific parameters, which were mainly in the context of detecting somatic mutations^22^.

This current study is a systematic analysis of tumour purity across multiple cancer types using four different methods and an additional consensus method. We distinguished between the effects of intrinsic and extrinsic factors on tumour purity and analysed the implications of these effects on clinical and molecular information. Intrinsic factors imply that purity levels are a characteristic of the tumour, and that purity variation results from clinical variability. In this case, purity should be associated with clinical information and outcomes. Extrinsic factors imply that purity is dependent on how a sample is collected. In this case, we expect only confounding associations with genomic reasoning such as clustering, correlating and differential analysis of tumour samples. When we adjusted gene expression analysis to account for purity estimations, we saw upregulation of immunotherapy-related T-cell activation pathway genes in several cancer types, which cannot be detected in traditional differential expression (DE). Our systematic analysis provides a comprehensive evaluation of the importance of accounting for tumour purity in cancer studies.

## Results

### Purity of TCGA tumour samples

**Figure 1:**
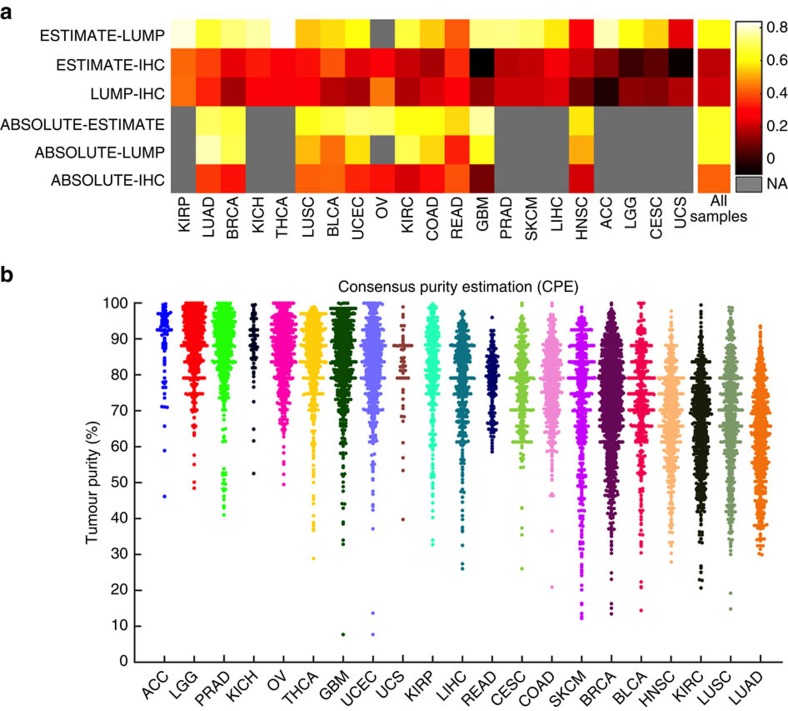
Tumour purity of TCGA cancers types. (**a**) Pairwise correlations between tumour purity methods used in 21 cancer types and all samples combined. Grey cells: data not available from both purity methods. Correlations between the genomic-based methods are high in most cancer types. Correlations with IHC are low, yet always positive. (**b**) Violin plots of CPE tumour purity in 21 cancer types. The cancers were ordered according to median purity.

We obtained gene expression profiles (RNA-seqV2), DNA methylation profiles (HumanMethylation450) and immunohistochemistry (IHC) analysis for 9,364 tumour samples and 1,958 adjacent normal samples across 21 solid tumour types from the TCGA repository (Supplementary Table 1). For each sample, we assigned purity estimates using four methods: ESTIMATE, which uses gene expression profiles of 141 immune genes and 141 stromal genes^6^; ABSOLUTE, which uses somatic copy-number data (estimations were available for only 11 cancer types)^7^; LUMP (leukocytes unmethylation for purity), which averages 44 non-methylated immune-specific CpG sites (Supplementary Fig. 1 and Methods); and IHC, as estimated by image analysis of haematoxylin and eosin stain slides produced by the Nationwide Children’s Hospital Biospecimen Core Resource. All estimates are in Supplementary Data 1. The purity estimates from the DNA, RNA and methylation-based methods had high concordance between most cancer types (Fig. 1a; Supplementary Fig. 2). The correlation of the three genomic-based methods with IHC was lower in all cancer types (Supplementary Fig. 3). However, in all assayed methods, the correlation coefficients with IHC were positive among all cancer types, suggesting that IHC provides a qualitative estimation of purity.

Next, we determined the average purity level of all associated tumour samples for each cancer type, according to each method. In accordance with previous results, there was high concordance among the DNA, RNA and methylation-based methods, and lower to no agreement with IHC (Supplementary Fig. 4). Average tumour purity across all samples from all cancer types was 81.1±13.9%, 76.1±16.1% and 75.7±21.2% (mean±s.d.) for ESTIMATE, LUMP and IHC, respectively. An exception was ABSOLUTE, with an average estimate of 62.3±19.9%. This difference is explained by methodological differences: while ABSOLUTE is a direct measure of the tumour cells in a sample, ESTIMATE and LUMP estimate purity indirectly by measuring immune and stromal counterparts in the sample. Thus, the difference in the average estimates is arguably due to the presence of non-immune and stromal cells in a sample, such as contaminating adjacent normal cells, which are not measured by ESTIMATE and LUMP.

We used these methods to derive a consensus measurement of purity estimations (CPE). CPE is the median purity level after normalizing levels from all methods to give them equal means and s.d.’s (75.3±18.9%). CPE is used in all analyses below, but results were consistent in the majority of methods when individually tested (presented in Supplementary Figs 1–15). Regardless of the method used, purity patterns differ by cancer type, with high purity (over >90%) in brain-originating tumours such as adrenocortical carcinoma (ACC) and lower-grade glioma (LGG) and low purity (<70%) in cancers resulting from chronic mutagenic exposures, such as lung adenocarcinoma (LUAD), squamous cell carcinoma and head and neck squamous cell carcinoma (Fig. 1b; Supplementary Fig. 5).

**Figure 2:**
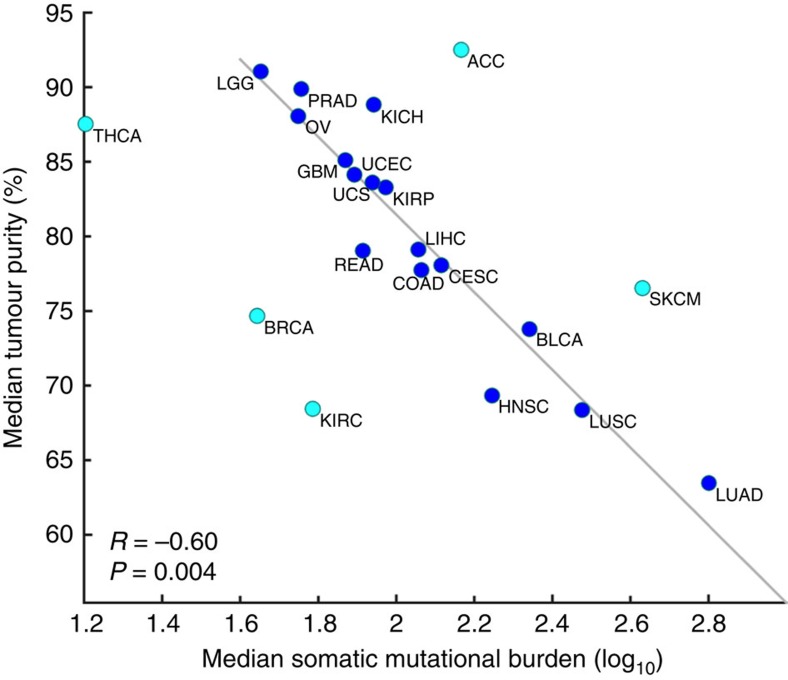
Tumour purity and mutational burden. Scatter plot of median number of mutations per tumour sample for each of the 21 cancer types (log 10 scale) versus median tumour purity as calculated by CPE. Pearson coefficient is presented. The least-squares line presented was calculated without the five outliers coloured in lighter blue.

This variation in purity levels resembles variation in mutational rates between cancer types, which are commonly associated with more robust response to therapies targeting immune checkpoint pathways^20^,^23^. We examined correlations between median purity levels and median mutational burden of cancer types, and found a significant association between them (Pearson correlation *R*=−0.60, *P*=0.004; Fig. 2; Supplementary Fig. 6). This association seems to be highly consistent throughout the range of mutational burden. The only cancer types not following this strong correlation were thyroid papillary carcinoma (THCA), breast invasive carcinoma (BRCA) and kidney renal clear cell carcinoma (KIRC), each with lower mutational burden than expected by purity; and ACC and skin cutaneous melanoma (SKCM), each being higher than expected. Interestingly, ACC and SKCM had the lowest missense:silent ratio of all cancer types, while BRCA had the highest, suggesting an underlying force in those cancer types that drives them away from the purity-mutational burden curve. An analysis restricted to the remaining 16 cancer types gave an almost perfect correlation (Pearson correlation *R*=−0.94, *P*=4.4e−8). However, it is important to note that we did not observe significant negative correlations in a sample-by-sample correlation in specific tumour types, suggesting that this association is a property of cancer types rather than individual patients.

Within cancer types, we found major differences between different samples. For example, in SKCM, 56 of the samples (11.8%) were highly purified (>90%), while 95 (20.0%) had poor purity (<60%). While only 1.9% of the samples had purity levels lower than the TGCA’s minimum of 60% by IHC, 40.3, 8.9 and 18.5% had low purity according to ABSOLUTE, ESTIMATE and LUMP, respectively.

We investigated different samples from the same patient to determine whether they were concordant. Across all cancer types, 37 patients were analysed twice from two different portions of the same sample. We observed high concordance between the samples (Pearson correlation *R*=0.73, Supplementary Fig. 7). This result persisted even when analysing cancer types separately. For example, purity from the 10 LUAD patients with two samples was highly correlated (Pearson correlation *R*=0.82). We concluded that differences in purity levels among cancer patients and cancer types are robust, consistent and specific to the tumour.

### Tumour purity versus clinical features and outcomes

**Figure 3:**
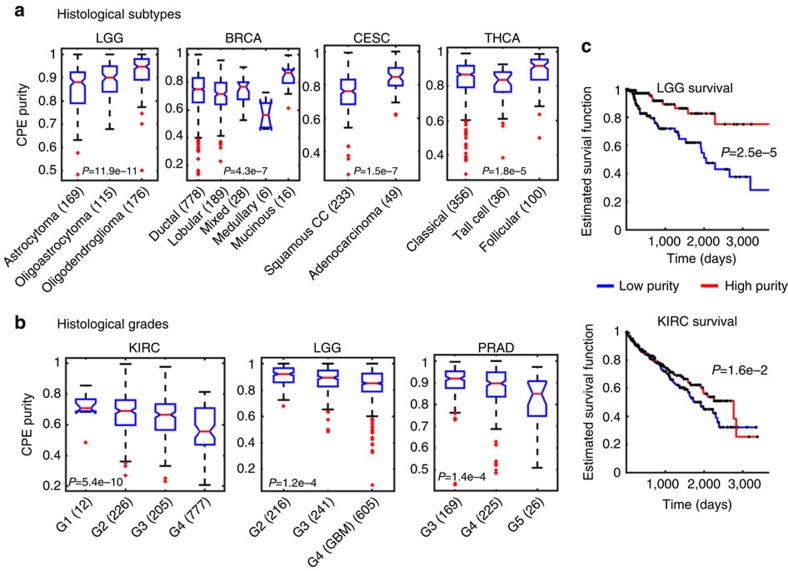
Tumour purity and prognosis. (**a**) CPE tumour purity in different histological subtypes in lower-grade glioma (LGG), breast (BRCA), cervix (CESC) and thyroid (THCA) tumour subtypes. Sample numbers are in parentheses. One-way analysis of variance (ANOVA) *P* values are presented. (**b**) CPE tumour purity levels in different histological grading methods. Histological grade is shown in kidney renal clear cell carcinoma (KIRC), LGG and prostate adenocarcinoma (PRAD). Breslow’s depth value grouped in stages is shown in melanoma (SKCM). In LGG, the purity level of glioblastoma (GBM), which is grade 4, is shown as a reference. In PRAD, the grade is of the primary pattern. One-way ANOVA *P* values are presented. (**c**) Kaplan–Meier survival plot in LGG and KIRC patients with low purity (3rd tertile) and high purity (1st tertile). Log rank *P* values are presented.

The observation that tumour purity was maintained in different samples from the same tumour suggested that purity is an intrinsic property of the tumour. We sought to explore whether it was associated with clinical features. We examined associations between purity levels and all available clinical features provided by TCGA in each cancer type. We analysed 722 clinical features, spanning 299 unique features (Supplementary Data 2). Generally, characteristics including sex, age, ethnicity, alcohol use and smoking were not associated with tumour purity. However, we detected 11 associations (false discovery rate <1%) with features characterizing the tumour, most prominently with histological tumour analyses in different cancer types. Histological subtypes, which are classified based on cell type and pattern, are frequently treated as similar entities of the same cancer type, although there are obvious differences in the tumour’s biological characteristics and prognosis. We observed differences in purity levels between the different histopathological subtypes of LGG, BRCA, THCA, and between cervical squamous and adenocarcinomas (CESC; Fig. 3a; Supplementary Fig. 8a–d). We additionally observed a consistent decrease in purity as tumour grade progressed in KIRC and LGG, which is consistent with the lower purity of glioblastoma (GBM) samples (grade 4), and in the primary grade of prostate adenocarcinoma (Fig. 3b; Supplementary Fig. 8e–g). In LGG, we found differences in purity at different tumour locations (Supplementary Fig. 8h). In BRCA, we also found differences between oestrogen receptor-positive and -negative samples (Supplementary Fig. 8i). The only significant non-pathological associations of purity we found were a history of thyroid gland disorder in THCA and presence of IDH1 mutation in LGG (Supplementary Fig. 8j–l). The latter association likely results from the fact that LGG tumours with wild-type IDH1 are molecularly and clinically similar to GBM^24^, which have lower purity levels. Divergent purity levels were found prominently in pathologic diagnoses, and moreover, the lack of association between purity and patient characteristics suggests that purity differences is at least not an intrinsic characteristic, but a result of the sampling by the surgeon and the level of difficulty separating it from its environment.

We employed a Cox proportional hazard regression analysis to test for association between purity and survival time. We found associations with purity with three methods in KIRC and LGG (Fig. 3c; Supplementary Table 2). As described, purity in LGG samples differed between histological subtypes. Survival analysis was consistent with these findings, as astrocytomas tend to have poorer prognosis than other subtypes^25^. This result could also be explained by clinical outcomes associated with IDH1 mutation^24^, which is also associated with purity, as shown above. Our observation in KIRC may explain prognosis for this cancer as well, as we found lower purity in higher-grade tumours. These explanations reinforce our claim that purity differences are extrinsic.

### Tumour purity confounds genomic analyses

We next examined the confounding effect of tumour purity on genomic analyses. We divided this effect into three commonly used bioinformatics methods: correlation, clustering and differential analysis. Our presentation focuses on gene expression profiles, but all the analyses hold to the same extent in other genomic measurements.

**Figure 4:**
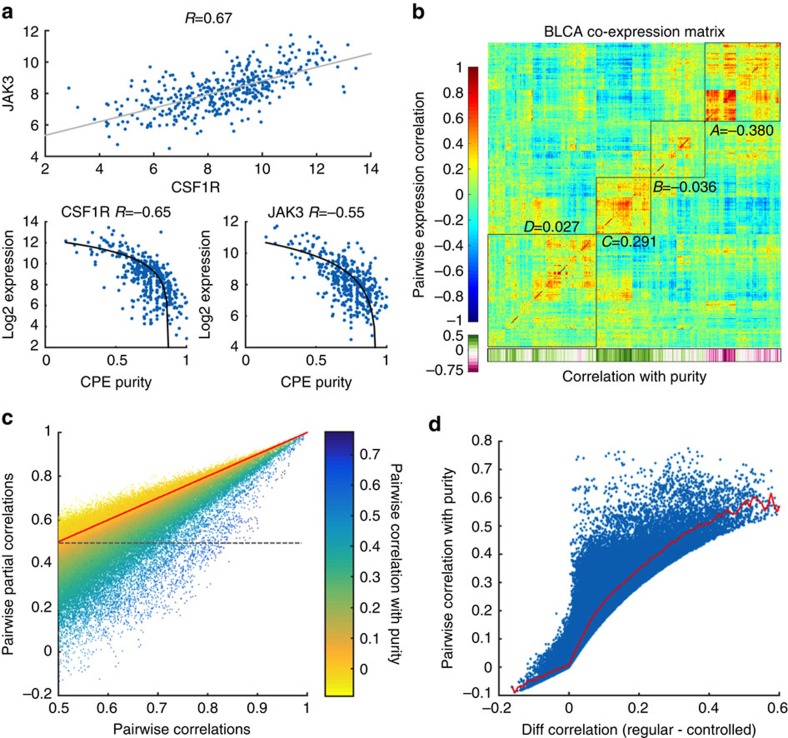
Tumour purity confounds co-expression analysis. (**a**) The problem of co-expression without accounting for sample purity. Top panel: correlation of expression between colony-stimulating factor 1 receptor (CSF1R) and Janus kinase 3 (JAK3) in bladder urothelial carcinoma (BLCA). Lower panels: high linear correlation between tumour purity and expression of those genes. The *y* axis is in log scale, and the fitted line is in log scale accordingly. There is no known interaction between these genes. (**b**) Co-expression matrix of top 5,000 genes according to gene expression s.d. Cell(*i*,*j*) is the Spearman coefficient between expression of gene *i* and gene *j*. Genes were clustered according to the Euclidean distance between them. Bottom vector: coloured by the correlation of the genes with purity. The four major clusters are boxed; average correlation of genes with purity in the cluster is shown. Group A is highly enriched with genes negatively correlated with purity. Group C has only genes positively correlated with purity. (**c**) Scatter plot of co-expression correlations (*x* axis) versus partial correlation of co-expression controlling for CPE purity levels (*y* axis) in all 21 cancer types. Analysis was restricted to the top 1,000 genes according to gene expression standard deviation in each cancer type, and the plot shows correlations with a Spearman coefficient >0.5. The colours correspond to the multiplication of the correlation of the co-expressed genes with purity. (**d**) Scatter plot of the difference in correlation between regular co-expression and purity controlled pair of genes (*x* axis) versus the pairwise multiplication of the co-expressed genes with purity. Red line: kernel smoothing regression of the data.

Correlative analyses are widely applied to genomics in the study of cancer. One key approach is the gene co-expression network, which assigns a score to a pair of genes based on their co-expression frequencies in different samples. Co-expression networks have been used extensively in cancer studies, with an aim to unravel hallmark pathways and prioritize novel candidate genes^26^. We found that identifying co-expression networks from genomics data without accounting for tumour purity is problematic. Gene expression profiles from bladder carcinoma illustrate the problem. For example, expression levels of colony-stimulating factor 1 receptor (*CSF1R*) and Janus kinase 3 (*JAK3*), tyrosine protein kinases and known cancer-driver genes^27^, are highly correlated with each other (Spearman correlation *R*=0.67, *P*<1e−20). Thus, one might suggest a shared co-expression network between them, which would be a novel finding. However, this correlation likely results from the high correlation of both genes with tumour purity (Fig. 4a).

We extended this observation to all available gene pairs. Strikingly, we found that the strongest gene networks, that is, groups of genes with correlated expression profiles, were composed of genes highly associated with purity (Fig. 4b; Supplementary Fig. 9). Group A, which contains 25.7% of the genes, was enriched with 60.0% of all co-expressing gene pairs (Spearman coefficient |*R*|>0.5), but also with genes negatively correlated with purity (91.1% of genes with *R*<−0.3). In total, 49.7% of co-expressing genes were between genes that were both correlated with purity (|*R*|>0.3), compared with an expected ratio of only 0.6%. As expected, the group A gene ontology annotations were enriched with terms related to the immune system, but also with other terms such as extracellular matrix organization and other cellular functions (Supplementary Table 3). Group C, on the other hand, contained only genes positively correlated with purity. Those genes did not seem to share specific gene ontology annotations. While genes in both groups may be part of a shared co-expression network, the above analysis demonstrates that a correlation between them may be explained in large part by tumour purity. We attempted to address this bias by applying partial correlations with controlling for tumour purity in the co-expression analysis. The number of pairwise co-expressions in bladder carcinoma decreased by 39.7%, and the fraction of co-expressions between purity-associated genes decreased by 58.4%. Overall in all 21 cancer types, we observed a 21.0% decrease in the number of pairwise co-expressions when controlling for purity (Fig. 4c; Supplementary Fig. 10), and a decrease of 48.7% of co-expressions when both genes are correlated with purity. This decrease was tightly correlated with the pairwise correlation of the genes with purity (defined as the multiplication of the coefficients of the correlation of expression with purity between the co-expressing genes). For every 0.1 increase in the level of pairwise correlation with purity, we observed a 0.1 correlation decrease (Fig. 4d). We concluded that naive correlation between genomic profiling measures gives results that are highly confounded by tumour purity. We suggest that future co-expression analyses should employ partial correlation analysis by adjusting for tumour purity.

**Figure 5:**
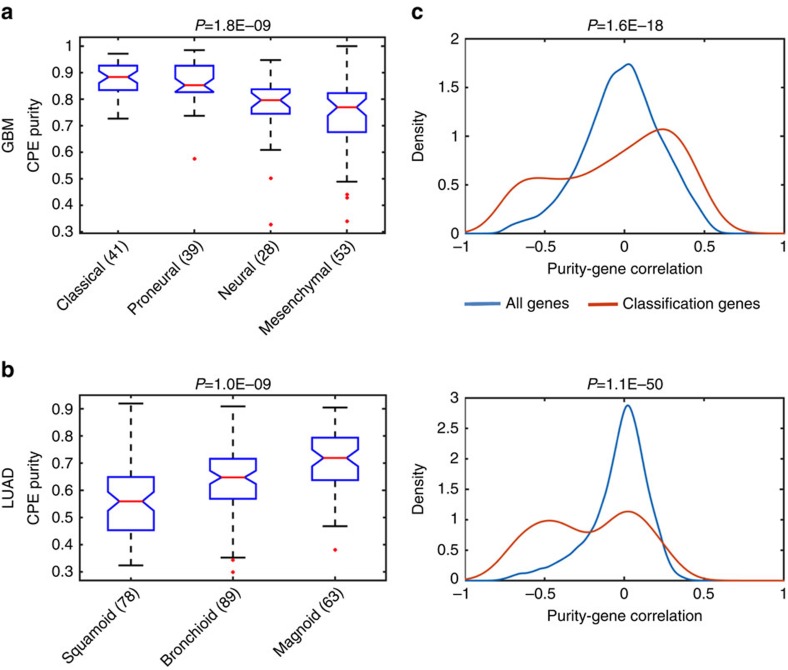
Tumour purity confounds molecular subtyping. (**a**) Boxplot of molecular subtypes as a function of tumour purity in glioblastoma (GBM). The numbers of samples associated with the subtypes are in parentheses. One-way analysis of variance *P* value is presented on top. The central red mark is the median; the edges of the box are the 25th and 75th percentiles. (**b**) Same as **a** for lung adenocarcinoma samples (LUAD). (**c**) Distributions of the Spearman coefficient of genes with purity in GBM (up) and LUAD (down). Blue curves: distributions for all genes; red curves: the 840 and 506 genes used for classifying the subtypes in GBM and LUAD, respectively. Kolmogorov–Smirnov *P* values are on top.

The subclassification of cancers based on genomic measurements has been a fundamental part of cancer research and therapeutics development in recent years. Numerous publications have applied molecular subtyping methods in different cancer types^28^,^29^, and have shown its power in facilitating precision medicine^30^. It should be emphasized that employing genomic measurements for subtyping tumours is distinct from histological subtyping by visual analysis, though there have been attempts to consolidate these two approaches. This study highlights the risk of confounding potential tumour purity when applying unsupervised clustering for molecular subtyping. In three cancer types—breast, GBM and LUAD—the molecular subtypes and the subtyping method based on gene expression profiles are widely accepted, and in all three, we detected discrepancies in purity among subtypes. Four molecular subtypes of GBM have been proposed: classical, neural, proneural and mesenchymal^31^. Purity analysis on centroids of 840 genes revealed consistently lower purity in the mesenchymal and neural subtypes (Mann–Whitney *U*-test *P*=1.8e−9; Fig. 5a; Supplementary Fig. 11). Three LUAD subtypes have been proposed: magnoid, bronchoid and squamoid^32^. The classification utilizes centroids of 506 genes^33^. Again, purity is a dominant factor in distinguishing the three subtypes (Mann–Whitney U-test *P*=1.0e−9; Fig. 5b; Supplementary Fig. 12). We suspected that associations between purity and molecular subtyping resulted from use of unsupervised clustering techniques, which emphasizes genes that are associated with purity. Thus, 47.1% and 45.4% of the genes used for subtyping in GBM and LUAD, respectively, were correlated with purity (|*r*|>0.3) compared with 21.2 and 10.7% of all genes (*P*=1.6e−18 and *P*=1.1e−50, Kolmogorov–Smirnov test; Fig. 5c). It should be noted that the differences in purity between subtypes might still be genuine and intrinsic characteristics of the subtypes. We suspect that this is the case in the molecular subtypes of BRCA. Our analysis detected differences in purity levels among the PAM50 molecular subtypes of BRCA^34^ (Supplementary Fig. 13); however, these differences are consistent with our finding of differences in oestrogen receptor status as obtained from pathologic analysis (Supplementary Fig. 8i). In the other cancer types, where classification is based on unsupervised clustering techniques and there are currently no non-molecular factors that distinguish subtypes, the confounding effect of tumour purity is alarming. We hypothesize that clustering with expression levels adjusted for purity will point to a different subtyping strategy for these samples.

**Figure 6:**
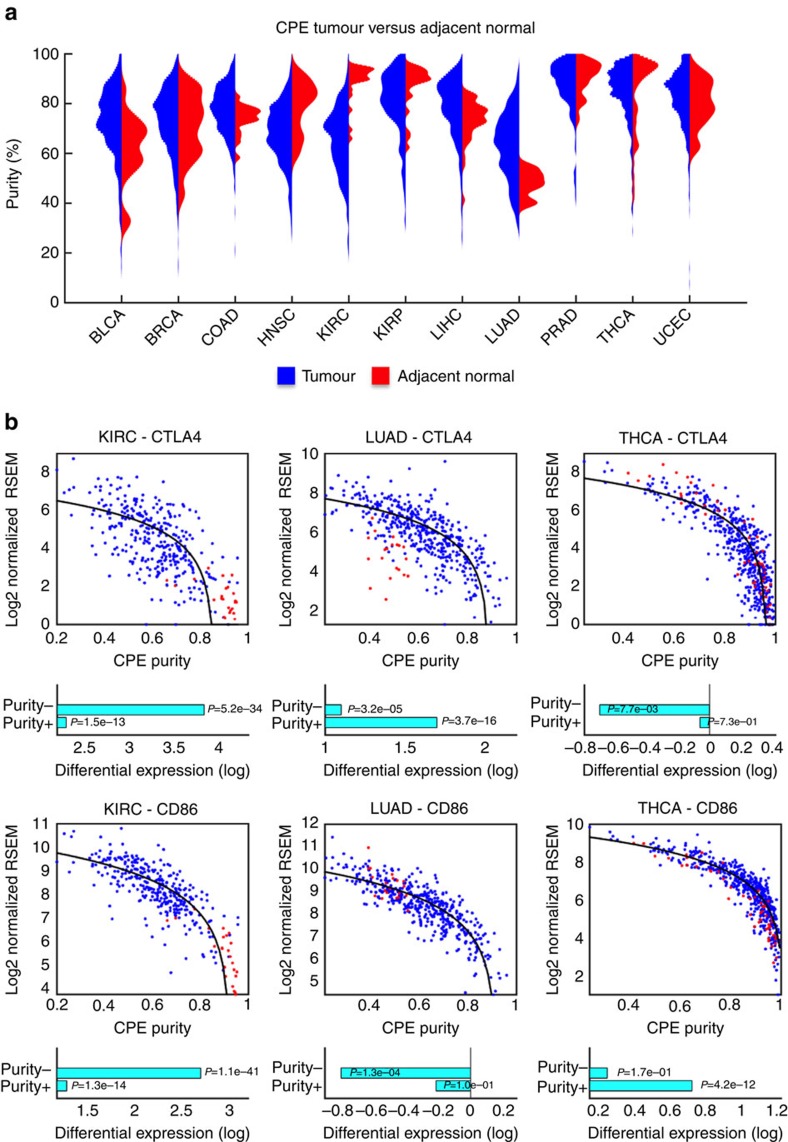
Differential expression analysis adjusted to tumour purity. (**a**) Violin plots of CPE purity in 13 TCGA types. Blue distributions: tumour samples; red distributions: non-tumour adjacent normal tissue. (**b**) CTLA-4 and CD86 expression profiles (*y* axis) versus CPE purity levels (*x* axis) in kidney renal clear cell carcinoma (KIRC), lung adenocarcinoma (LUAD) and thyroid carcinoma. Black curve: linear fit for purity and expression. For presentation purposes, the *y* axis uses a log2 scale. Bottom vertical bars: differential expression levels in log2 scale as calculated by DESeq2 in a traditional analysis (purity−) and adjusted analysis (purity+).

Last, we analysed the confounding effect of purity on DE analysis. Identifying differentially expressed genes in tumours is an important tool for studying tumorigenesis, and has been routinely applied to identify diagnostic and prognostic markers and therapeutic targets. We use the term ‘purity’ in a broad sense to define the proportions of non-immune counterparts in the sample, which can be calculated for both non-cancer and cancer samples, and can be estimated using ESTIMATE and LUMP. We applied a consensus estimate based on these two methods on normal samples of 13 cancer types with sufficient normal material (‘normal’ describes adjacent non-tumour samples). We found high concordance between the two methods in all cancer types except in LUAD (Supplementary Fig. 14a). We also observed high concordance between average purity estimates of TCGA normal samples and purity estimates of equivalent tissues taken from the Genotype-Tissue Expression project (Supplementary Fig. 14b)^35^. We found substantial differences in purity levels among different tissues and among different samples from the same tissue. Moreover, in several cancer types, we observed immense discrepancies between tumours and adjacent normal tissue (Fig. 6a; Supplementary Fig. 14c,d). For example, purity in normal kidney samples was, on average, 28.3% higher than the KIRC cancer samples. On the other hand, purity in normal lung samples was, on average, 26.7% less than in the lung squamous cell carcinoma cancer samples.

We used the DESeq2 package^36^ to apply DE analysis to RNA-seq counts of tumour and normal samples across a dozen cancer types with sufficient normal tissue for sampling. We compared our findings with a DE analysis designed to include purity estimates, which is equivalent to adjusting gene expression by purity. This comparison found numerous marked differences in relative expression levels. Many genes were differentially expressed before purity adjustment, but no differences between cancer samples and controls were seen after adjustment. Some genes even changed state from up- to downregulation or the other way around. Most importantly, we found differentially expressed genes after adjustment that had not been identified before. Figure 6b illustrates expression patterns of the immunotherapy target cytotoxic T-lymphocyte-associated protein 4 (CTLA4) and its ligand, CD86 (also known as B7.2) in traditional and adjusted DE analyses in three cancer types. Standard DE analysis labelled both genes as highly upregulated in KIRC samples. However, most of the difference from healthy samples could be ascribed to differences in purity. In LUAD, on the other hand, CTLA4 was detected as upregulated only after accounting for purity, while the downregulation of CD86 was again a byproduct of purity. In THCA, this trend was reversed: CTLA4 seemed downregulated, until DE adjustment, while CD86 was only detected as upregulated after adjustment.

On average, 13.7% of the genes originally considered as DE were lost, and 11.0% of genes were newly detected as DE after adjustment (Table 1; Supplementary Fig. 15). By ranking all genes by DE *P* value, we extracted genes that would have been missed in traditional analysis (Supplementary Data 2). We used Ingenuity Pathway Analysis^37^ to identify enriched pathways of for genes that after adjustment were ranked twice as high as before adjustment; this analysis revealed significant enrichments of many immune-related pathways in different cancer types (Fig. 6; Supplementary Data 3). Notably, the analysis highlighted different T-cell activation pathways in different cancer types, particularly the CTLA4, CD28 and iCOS-iCOSL signalling pathways in T cells, which are the key pathways in anti-CTLA-4 immunotherapy treatments^38^. As illustrated in Fig. 6b, genes in these pathways were prone to being ignored in traditional gene expression analysis, as their expression was masked by sample heterogeneity. We propose that considering tumour purity in DE analysis should be an integral tool for the discovery of novel genes and pathways altered in tumorigenesis.

## Discussion

This study of tumour purity found major differences in tumour purity levels (Fig. 1b). The proportion of healthy epithelial cells in tissue samples also differed between tissue types, so it is not surprising to find differences between cancer types (Fig. 6a). However, as shown in our results, there were major differences between purity of healthy samples and cancer samples from the same tissue type, suggesting a role for the microenvironment in the malignancy status quo. An alternative hypothesis is that the spread of the different types of tumours makes it harder or easier to distinguish cancer cells from the environment. This discrepancy was also found between samples from the same cancer type, whether differences between samples were intrinsic characteristics of the tumour or variations in the sample collection methods.

We found evidence for both possibilities. On one side, we did not find major clinical differences between patients in spite of varying tumour purity levels. The dominant clinical differences we did find were with pathological diagnosis or related to it, which is somewhat analogous to cancer-type differences (Fig. 3). Alternatively, samples from the same patient did have high concordance, suggesting that this finding is patient specific and an intrinsic characteristic of the tumour (Supplementary Fig. 7). However, one can argue that according to the type and grade of the tumour, it is difficult to distinguish tumorous from non-tumour tissue in all regions of the tumour. Another finding reported here was a strong association between tumour purity and mutational burden (Fig. 2). An inflammatory microenvironment is known to increase mutation rates^39^; thus, it is possible that our findings result from a negative correlation between purity and an inflammatory microenvironment, which in turn may strengthen the argument of intrinsic effects of purity.

Answering this question is important for understanding clinical properties of the tumour, but it is also important for understanding the tumour’s molecular properties. This study has shown the confounding effect of tumour purity on various types of analyses of molecular data sets. If differences between samples from the same cancer type can be attributed to the methods and skills of the sample collector, purity may be a major confounder in all ‘omics’ analyses, and should be accounted for in these studies. Alternatively, if differences are intrinsic to the samples, it is still important to determine which results in molecular analyses are related to differences in overall purity and which are not. For example, if a gene is designated as overexpressed in a subset of samples, is this finding due to variation in its regulation or to an increased proportion of cell types where expression is regular? We have highlighted the confounding effect of tumour purity in a basic and common bioinformatics toolset, co-expression analysis (Fig. 4) and molecular subtyping (Fig. 5). False interpretations in these analyses due to divergent tumour purity levels may have a negative effect on our understanding of cancer biology and on our ability to create treatments. We urge future cancer-related analyses of genomic data sets to account for purity levels.

**Figure 7:**
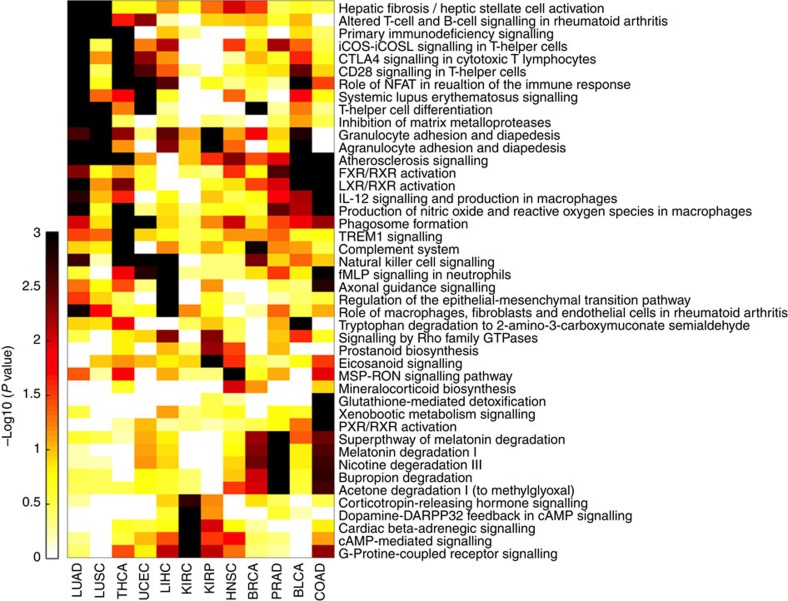
Enriched pathways of differential expression adjusted for tumour purity. Pathway analysis of genes whose ranks advanced significantly in purity+ compared with purity−. The plot shows pathways that were enriched in at least one of the cancer types. Black: highly enriched; white: no enrichment. Analysis was performed using Ingenuity Pathway Analysis.

In this report, we have focused on analysing clinical and molecular associations with the whole microenvironment, without referring to specific cell types and their proportions in a tumour. In recent years, researchers have developed many deconvolution methods to tackle this problem using gene expression profiles^16^,^40^,^41^,^42^,^43^. However, this problem is significantly more complex. We have shown variation between methods on the relatively simple problem of tumour purity. Assessing the specific proportion of dozens of cell types, some at very low abundance, is a difficult task, and published methods have yet to be validated in large-scale analyses. Further analyses are needed to discriminate between the effects of specific cell types in clinical and molecular data sets. However, although the obvious limitation, here we demonstrated how accounting for tumour purity as a whole reveals differentially expressed pathways that are now appreciated to be highly important to tumorigenesis (Fig. 7). These T-cell activation pathways, which are being exploited in different immunotherapy methods, are masked using traditional DE analysis, probably because of heavy infiltrations of immune cells in the tumour. Adjusting expression levels to purity estimates is a powerful computational tool to detect masked pathways, and can have an important role in discovering novel tumour pathways and developing novel therapeutics.

In conclusion, we have shown that the influence of tumour purity on the results of genomic analyses is much stronger than previously appreciated, and ought to be included as a covariate in any future analysis. Tumour purity differences resulting from sampling variation exceed intrinsic individual differences. Lower purity samples, by influencing genomic data, may make precision medicine efforts more challenging. We urge cancer researchers and clinicians to take tumour purity into account when analysing genomic data from patient samples.

## Methods

### Data sets

We accessed the TCGA data portal and downloaded level 3 RNA-seq profiles (RNAseqV2 normalized RSEM), level 3 HumanMethylation450 profiles, slide analyses, Mutation Annotation Format files, and clinical data for 21 solid human cancers and matched normal samples (https://tcga.data.nci.nih.gov/tcga/dataAccessMatrix.htm, download in February 2015). ESTIMATE scores were calculated using the ESTIMATE R package, and purity was estimated using the formula described in Yoshihara *et al*.^6^. ABSOLUTE levels for 11 cancer types were downloaded from synapse.org. We also attempted to calculate ABSOLUTE levels for missing data using the R package; however, our results were substantially different from the published estimates. ABSOLUTE data were calculated using segmented allelic data, which is not publicly available, and also require a manual parameter selection to ensure the best solution from several possible ones. Molecular subtypes were acquired from the UCSC Cancer Genome Browser (http://genome-cancer.ucsc.edu).

### Leukocytes unmethylation to infer tumour purity analysis

We obtained DNA methylation profiles (HumanMethylation450) for 10 immune cells (whole blood, peripheral blood mononuclear cell, granulocytes, neutrophils, eosinophils, CD4+, CD8+, CD14+, CD19+ and CD56+ cells) with six replicates each^44^. We found 30,106 sites that were consistently unmethylated (<5%) in the 60 samples. Employing DNA methylation profiles of tumour samples obtained from TCGA, we also searched for sites that were averagely methylated (>30%) in all 21 analysed cancer types. This yielded a list of 174,696 sites. The intersection of both lists was 44 CpG sites. LUMP estimations are the average methylation levels of these sites divided by 0.85. Supplementary Fig. 1 shows the high concordance of this method’s estimates with those produced by another DNA methylation-based method^7^ and downloaded from synapse.org.

### Consensus purity estimation method

We arbitrarily chose to normalize the purity levels ABSOLUTE, ESTIMATE, LUMP and IHC using the combined average and s.d. from all methods (75.3±18.9%). The CPE method is the median purity from each method after normalization. Because some samples did not have measurements from all four methods, we restricted CPE to samples with at least two measurements. In DE analysis, CPE levels were based only on ESTIMATE and LUMP, which can estimate purity in non-tumour samples, and we used samples estimated by at least one of the methods.

### Associating purity with clinical features

We downloaded TCGA’s clinical information data for each cancer type. For each binary or categorical clinical feature, we used one-way analysis of variance to calculate *P* values of purity in each category. For continuous clinical features, we calculated the *P* value of the Spearman correlation. We repeated these analyses for each of the five purity methods. A false discovery rate of 1% was chosen as a threshold for significance. Cox proportional hazard regression was used to analyse prognosis.

### Differential expression analysis

The DESeq2 software requires raw counts as input. We downloaded raw counts for all TCGA tumour samples from the GEO repository (GSE62944), and used the protocol generated by contributors^11^ to calculate raw counts for an additional 704 adjacent normal samples. We then used the DESeq2 R package twice for each cancer type between tumour and normal samples, once with only the condition as a factor, and again using both condition and CPE purity level (based on only ESTIMATE and LUMP). Next, we ranked genes according to the DE *P* values. Genes were labelled ‘newly discovered’ if they were (1) significantly differentially expressed after adjustment, (2) moved forward in rank by a factor of at least 2 and (3) moved forward in rank by at least 200 genes.

## Additional information

**How to cite this article:** Aran, D. *et al*. Systematic pan-cancer analysis of tumour purity. *Nat. Commun*. 6:8971 doi: 10.1038/ncomms9971 (2015).

## Supplementary Material

Supplementary Information Supplementary Figures 1-15 and Supplementary Tables 1-3

Supplementary Data 1 Tumor purity estimates for TCGA samples. Tumor purity estimates according to four methods and the consensus method for all TCGA samples with available data.

Supplementary Data 2 Clinical features association with tumor purity.For each binary or categorical clinical feature we use one-way analysis of variance (ANOVA) to calculate p-values of purity (CPE) in each category. For continuous clinical features we calculate p-value of the Spearman correlation. We repeated these analyses for each of the five purity methods. False discovery rate of 1% was chosen as a threshold for significance.

Supplementary Data 3 Rank changes in differential expression post adjustment to purity. Genes differential expression that have made a significant leap forward in rank after adjustment to purity in each cancer type.

## Figures and Tables

**Table 1:** Comparison of traditional and purity-adjusted differential expression analyses.

	Upregulated		Specific		Downregulated		Specific		Rank change	
	Purity−	Purity+	Purity−	Purity+	Purity−	Purity+	Purity−	Purity+	Up	Down
BLCA	1,137	929	322	114	1,300	1,205	180	85	128	109
BRCA	2,673	2,780	194	301	1,938	1,847	142	51	177	75
COAD	1,685	1,591	167	73	1,786	2,069	55	338	208	53
HNSC	1,410	1,486	186	262	1,518	1,890	147	519	477	238
KIRC	2,975	2,542	805	372	1,743	1,338	593	188	943	1,152
KIRP	2,028	2,025	141	138	1,662	1,751	114	203	253	107
LIHC	2,329	2,572	111	354	1,013	1,054	55	96	217	45
LUAD	2,272	2,112	389	229	1,516	1,374	246	104	265	24
LUSC	2,258	1,801	621	164	1,341	992	381	32	212	14
PRAD	877	801	112	36	1,318	1,292	161	135	206	98
THCA	1,659	1,809	55	205	1,100	1,151	49	100	247	33
UCEC	2,491	2,429	334	272	1,856	1,746	198	88	135	83
Average	1,983	1,906	286	210	1,508	1,479	194	162	289	169

BLCA, bladder carcinoma; BRCA, breast invasive carcinoma; COAD, colon adenocarcinoma; HNSC, head and neck squamous cell carcinoma; KIRC, kidney renal clear cell carcinoma; KIRP, kidney renal papillary cell carcinoma; LIHC, liver hepatocellular carcinoma; LUAD, lung adenocarcinoma; LUSC, lung squamous cell carcinoma; PRAD, prostate adenocarcinoma; THCA, thyroid papillary carcinoma; UCEC, uterine corpus endometrial carcinoma.

The table shows the number of genes up- and downregulated in tumours compared with normal samples in traditional analysis (purity−) and when accounting for tumour purity (purity+). Specific columns show the number of genes found exclusively in each design. Rank change columns show the number of genes whose ranks changed due to purity analysis.
