# Corrigendum: Systematic pan-cancer analysis of tumour purity

**DOI:** 10.1038/ncomms10707

**Published:** 2016-02-05

**Authors:** Dvir Aran, Marina Sirota, Atul J. Butte

Nature Communications 6 Article number: 897110.1038/ncomms9971 (2015); Published 12042015; Updated 02052016

In the Results section of this Article, haematoxylin and eosin staining data from the TCGA repository are incorrectly referred to as immunohistochemistry data. All references to ‘immunohistochemistry' or ‘IHC' should read ‘haematoxylin and eosin staining' or ‘H&E staining', respectively.

