# Supplementary material for: Systematic pan-cancer analysis of tumour purity
Source: Nat Commun. 2015 Dec 4;6:8971. doi: 10.1038/ncomms9971 (PMC4671203; doi:10.1038/ncomms9971)
Supplement: Supplementary Information — Supplementary Figures 1-15 and Supplementary Tables 1-3 [file ncomms9971-s1.pdf]

Supplementary Figure 1: LUMP – Leukocytes unmethylation to infer tumor purity

A

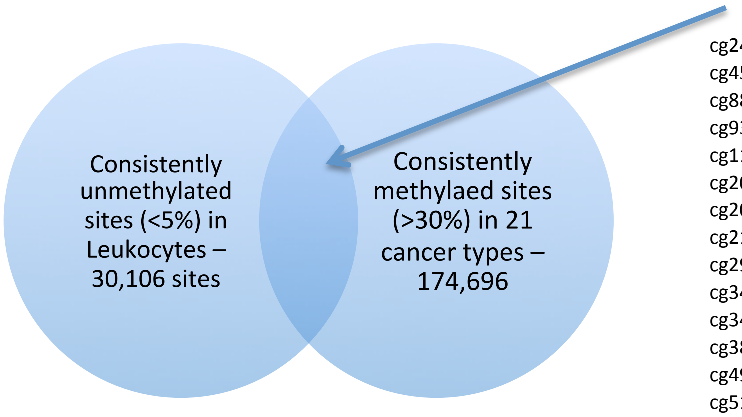

LUMP sites - 44 CpG sites

cg240653	cg5769344	cg19466818
cg450164	cg5798125	cg20170223
cg880290	cg7002058	cg20695297
cg933696	cg7598052	cg21164509
cg1138020	cg7641284	cg21376733
cg2026204	cg8854008	cg22331159
cg2053964	cg9302355	cg23114964
cg2167021	cg9606470	cg23553480
cg2997560	cg10511890	cg24796554
cg3431741	cg10559416	cg25384897
cg3436397	cg13030790	cg25574765
cg3841065	cg13912307	cg26427109
cg4915566	cg14076977	cg26842802
cg5199874	cg14913777	cg27215100
cg5305434	cg17518965	

$$LUMP = \min\left(\frac{\text{mean}(\text{LUMP 44 sites})}{0.85}, 1\right)$$

B

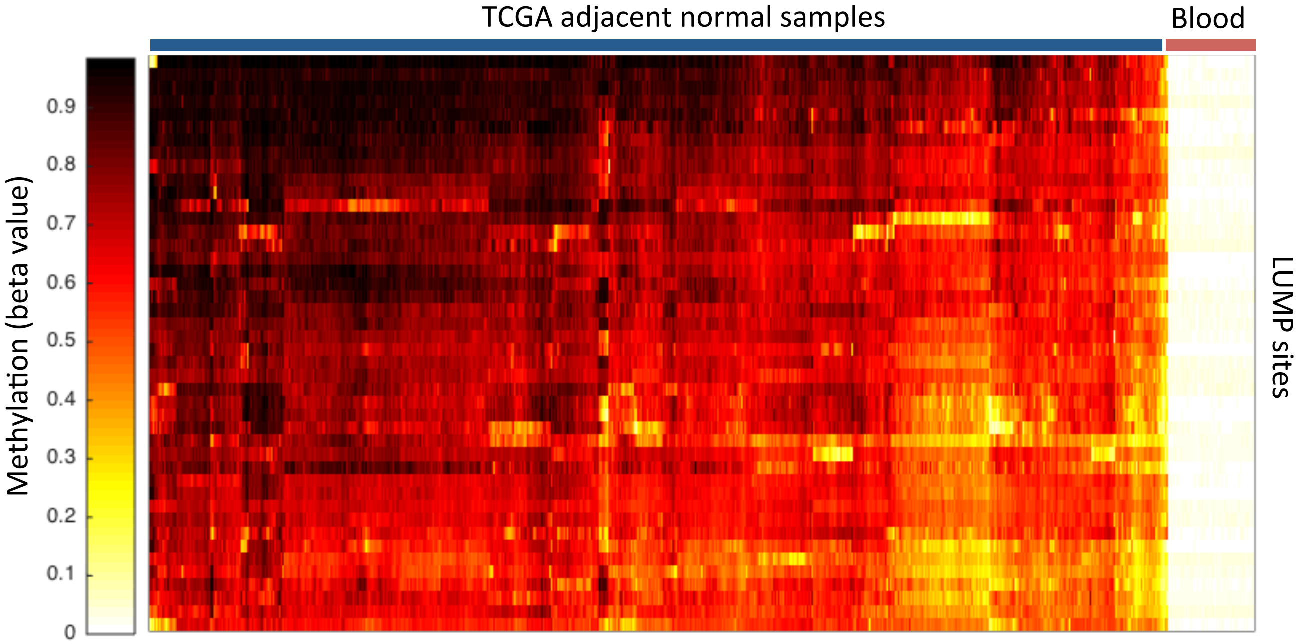

C

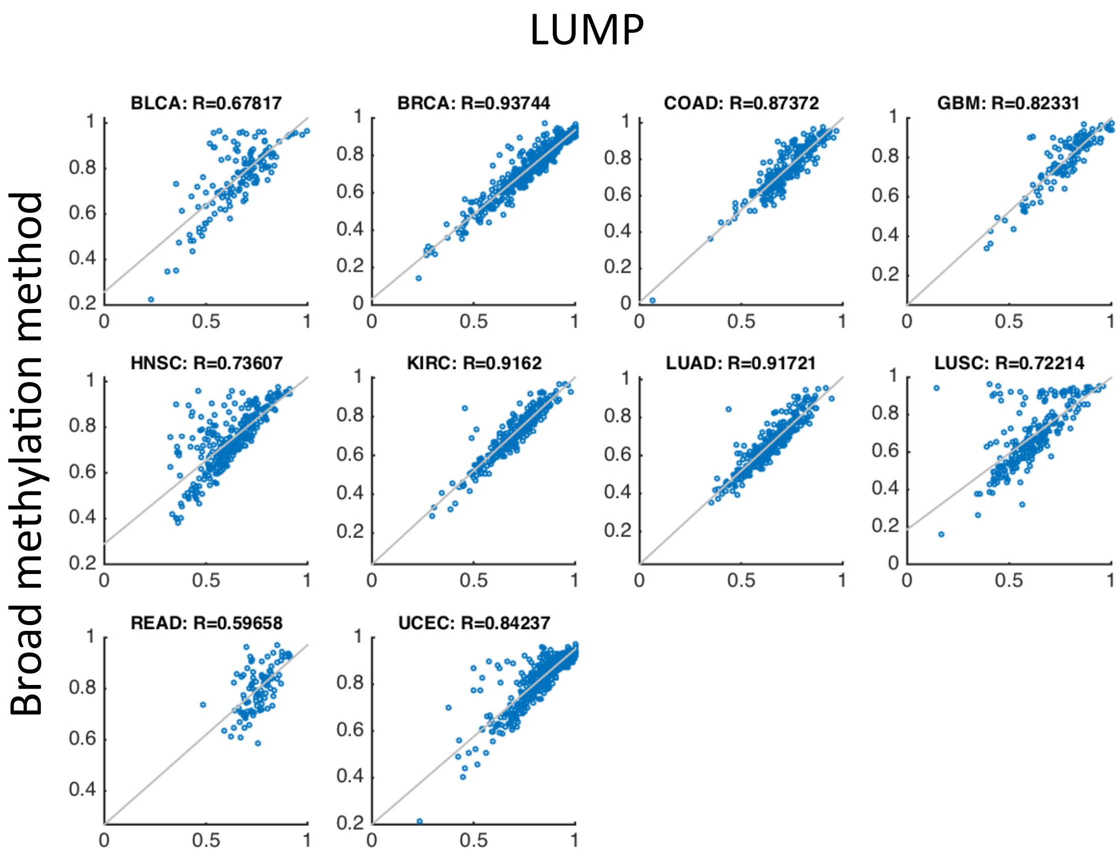

LUMP – Leukocytes unmethylation to infer tumor purity. A) We obtained DNA methylation profiles (HumanMethylation450) for 10 immune cells (Whole blood, PBMC, Granulocytes, Neutrophils, Eosinophils, CD4+, CD8+, CD14+, CD19+, CD56+) with 6 replicates each (Reinius et al. 2012). We first detected 30,106 sites that are consistently unmethylated (<5%) in all 60 samples. Employing DNA methylation profiles of tumor samples obtained from TCGA, we then searched for sites that methylated on average (>30%) in all 21 analyzed cancer types. This yielded a list of 174,696 sites. The intersection of both lists was 44 CpG sites which were further used to estimate purity in TCGA samples. **B)** Methylation beta values of 701 adjacent normal samples from TCGA and 60 immune cells for the 44 LUMP sites. The plot shows the low methylation of the LUMP sites in Leukocytes compared to non-blood tissues. **C)** Comparison of LUMP estimations and purity estimations produced by another DNA methylation methods for assesing purity (Carter et al. 2012) and downloaded from synapse.org. The list of sites used for this method was never published, and the estimations are available for 12 cancer types only. Here we show a simple DNA methylation method with high concordance to the previous method.

Supplementary Figure 2: Correlations between tumor purity genomic-based methods

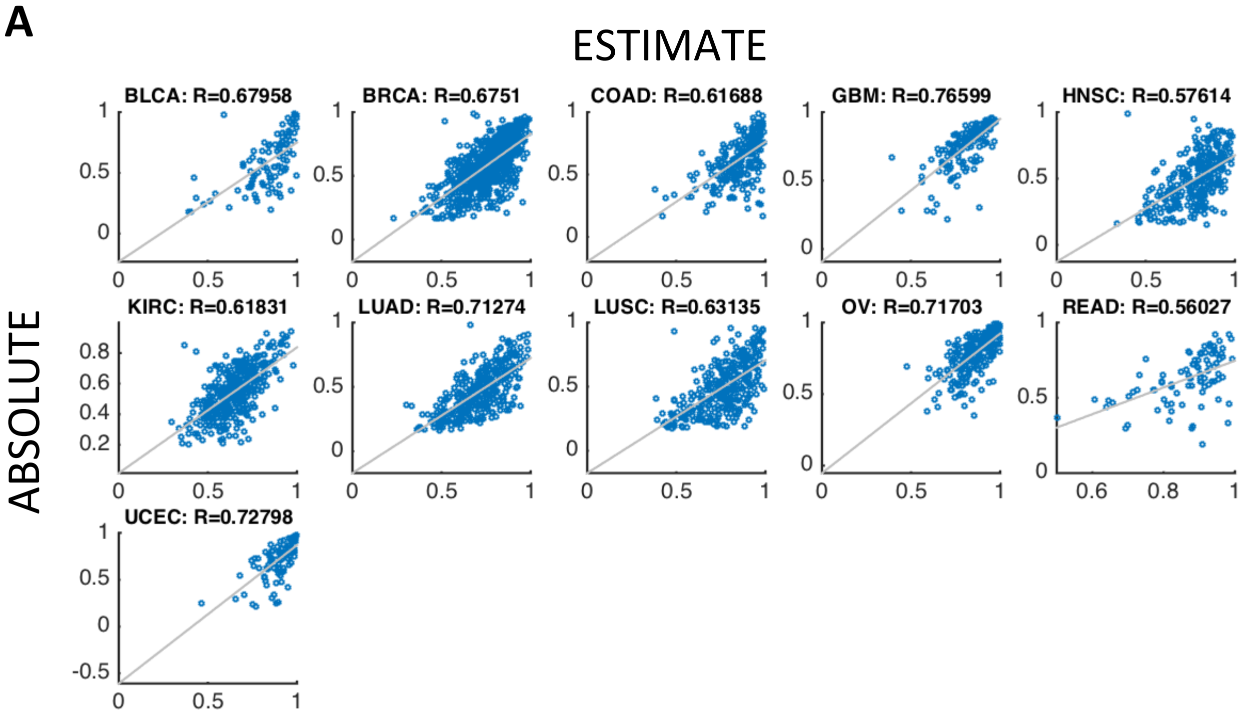

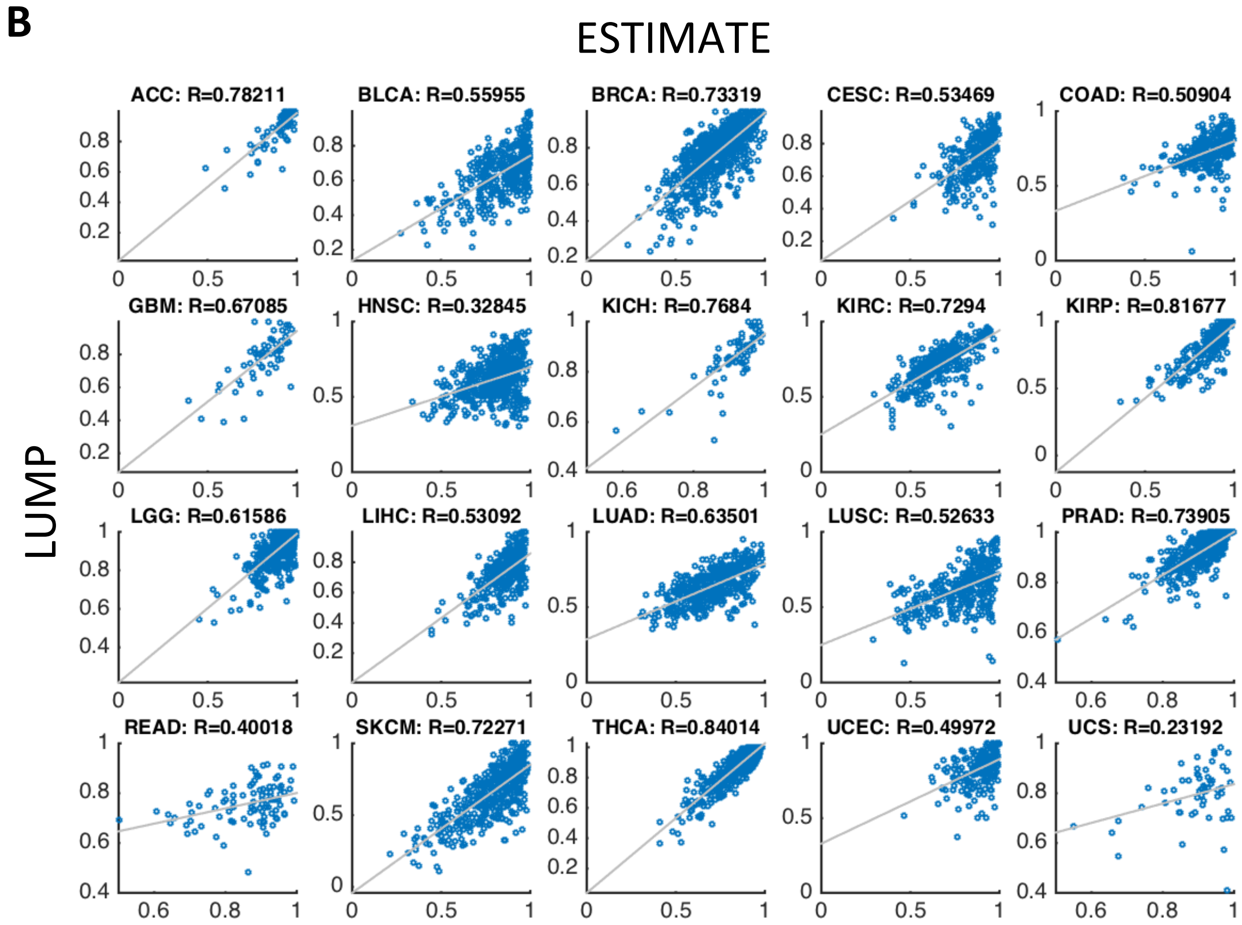

C

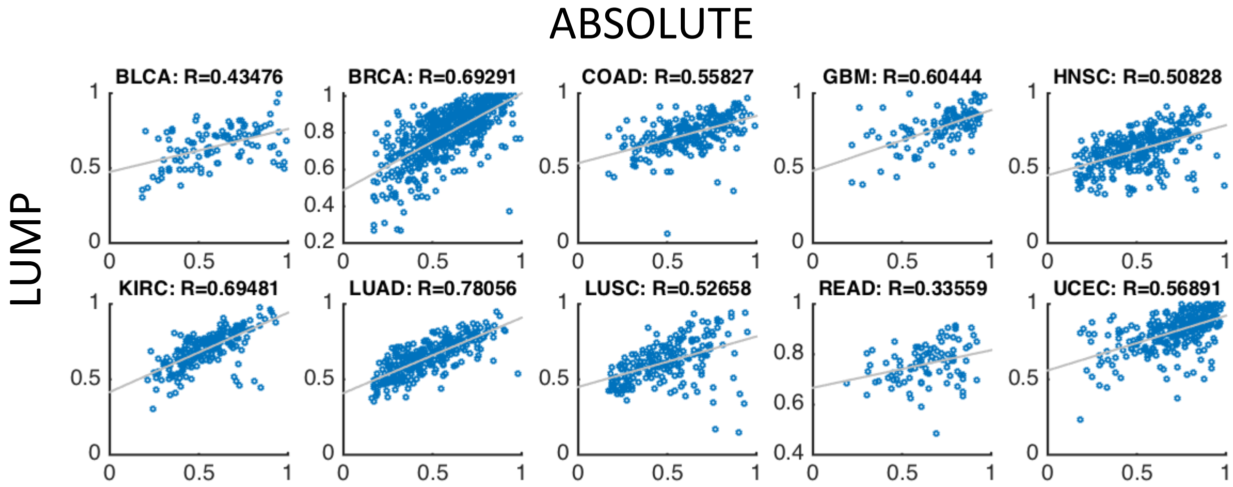

Correlations between tumor purity genomic-based methods. A) Scatter plots of tumor purity estimations in ESTIMATE vs. ABSOLUTE in 11 TCGA cancer types with available data. **B)** Scatter plots of tumor purity estimations in ESTIMATE vs. LUMP in 20 TCGA cancer types with available data. **C)** Scatter plots of tumor purity estimations in ABSOLUTE vs. LUMP in 20 TCGA cancer types with available data. Spearman coefficient is shown above each plot.

Supplementary Figure 3: Correlations between tumor purity genomic-based methods and immunohistochemistry (IHC) estimations

A

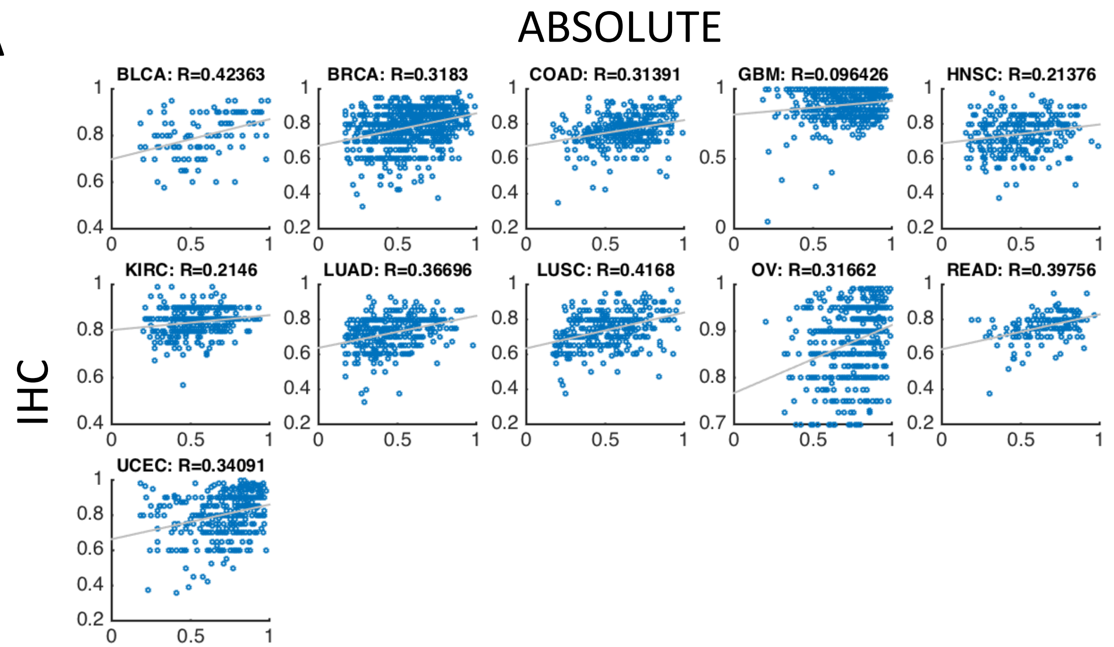

B

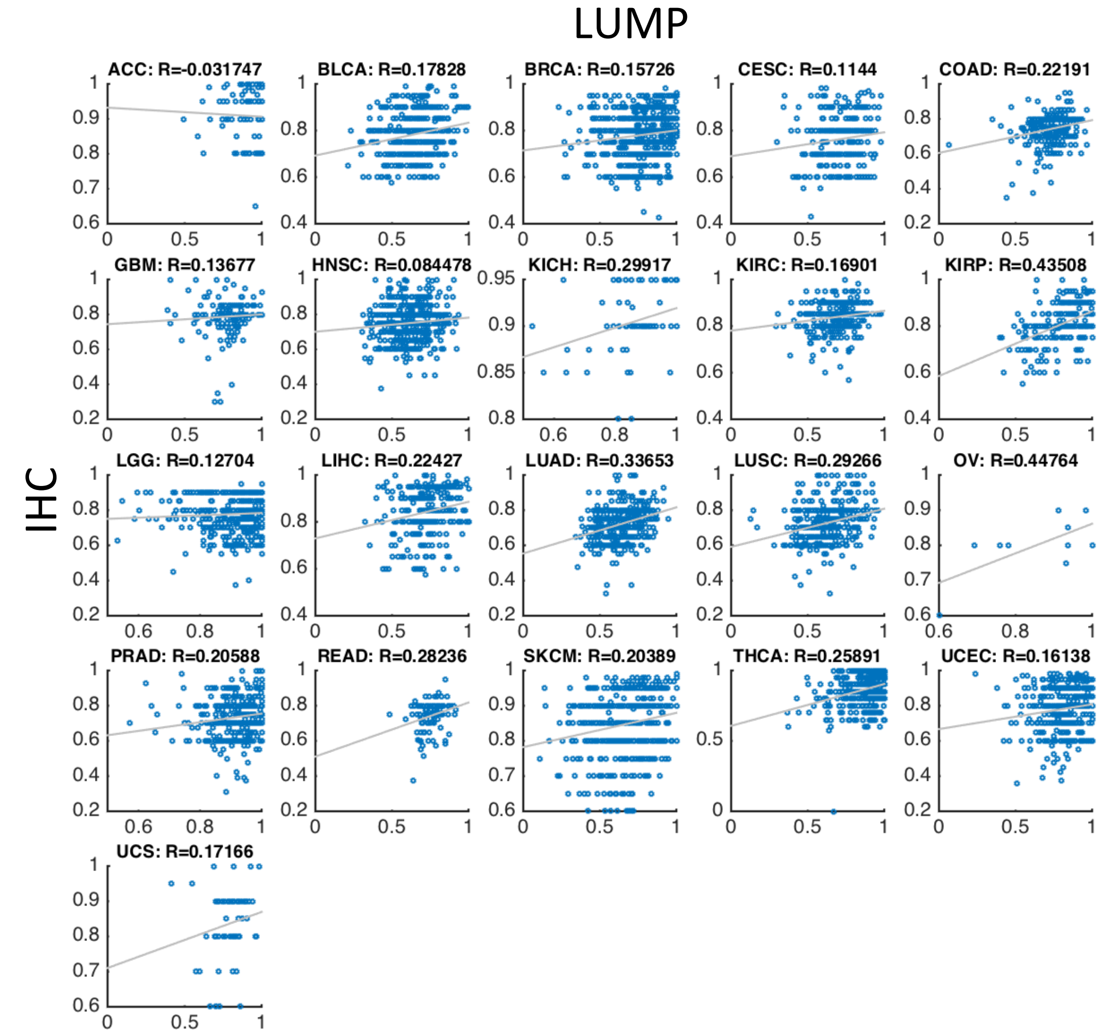

C

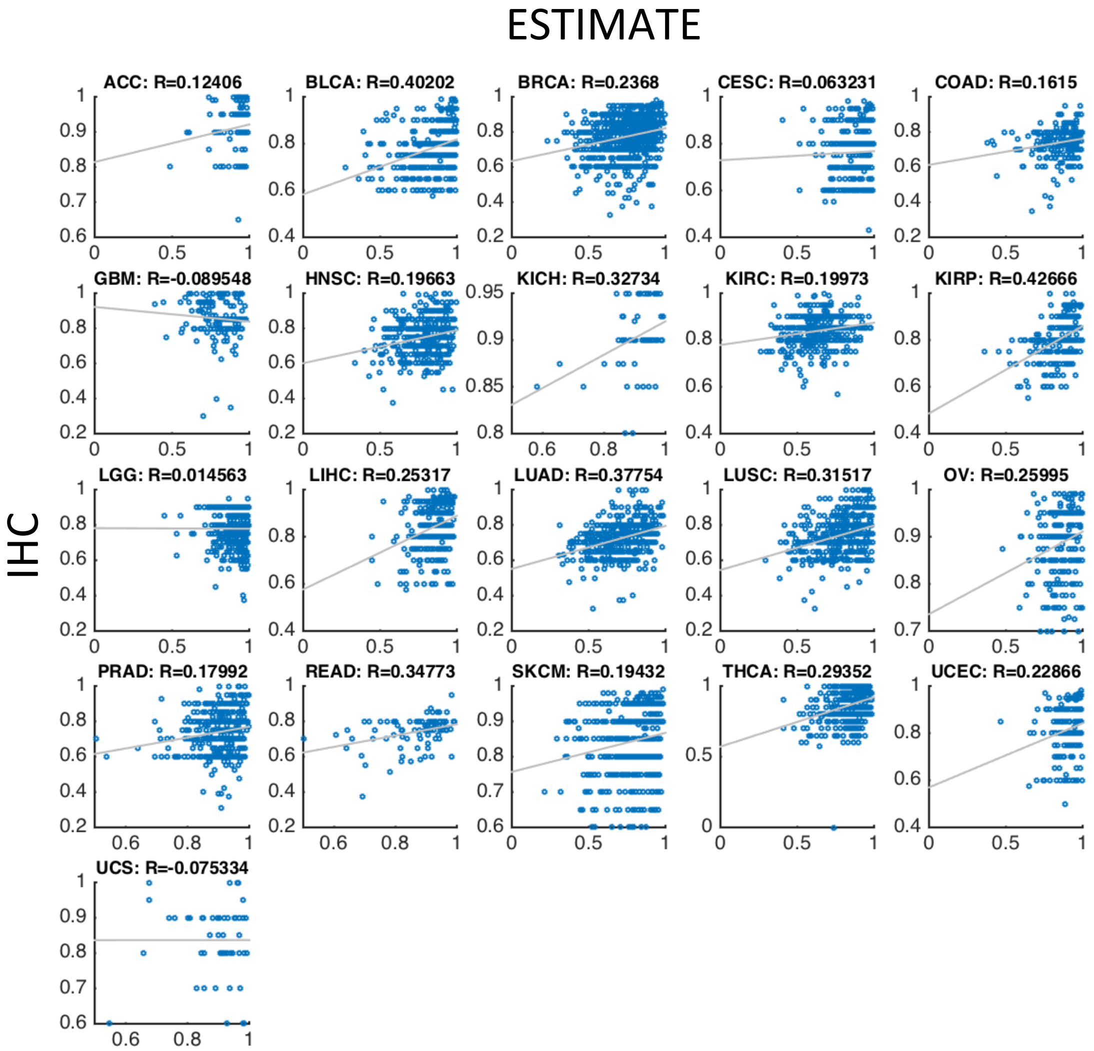

Correlations between tumor purity genomic-based methods and immunohistochemistry (IHC) estimations. A) Scatter plots of tumor purity estimations in ABSOLUTE vs. IHC in 11 TCGA cancer types with available data. **B)** Scatter plots of tumor purity estimations in LUMP vs. IHC in 21 TCGA cancer types with available data. **C)** Scatter plots of tumor purity estimations in ESTIMATE vs. IHC in 21 TCGA cancer types with available data. Spearman coefficient is shown above each plot. All correlations, except in UCS in ESTIMATE, are positive.

Supplementary Figure 4: Average purity levels for TCGA cancer types

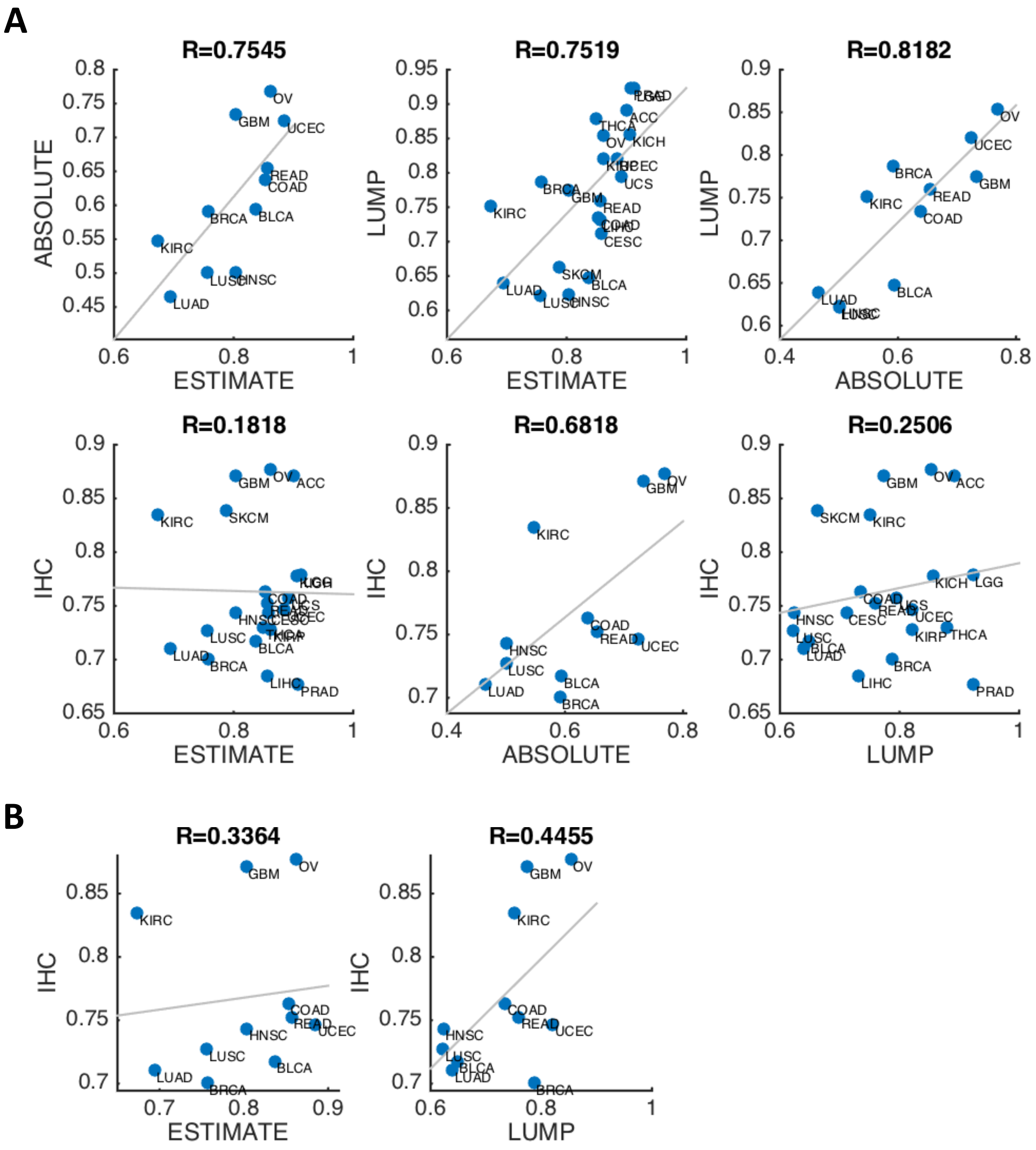

Average purity levels for TCGA cancer types. A) Correlation of cancer types averages between the four purity estimation methods. Spearman coefficient is shown above each plot. We observe high concordance between the genomic-based method, high concordance between ABSOLUTE and IHC, and low to none correlation of ESTIMATE and LUMP with IHC. **B)** Same, but only for cancer types available in the ABSOLUTE method. The correlation between LUMP and IHC is significantly better.

Supplementary Figure 5: Tumor purity of TCGA cancers types

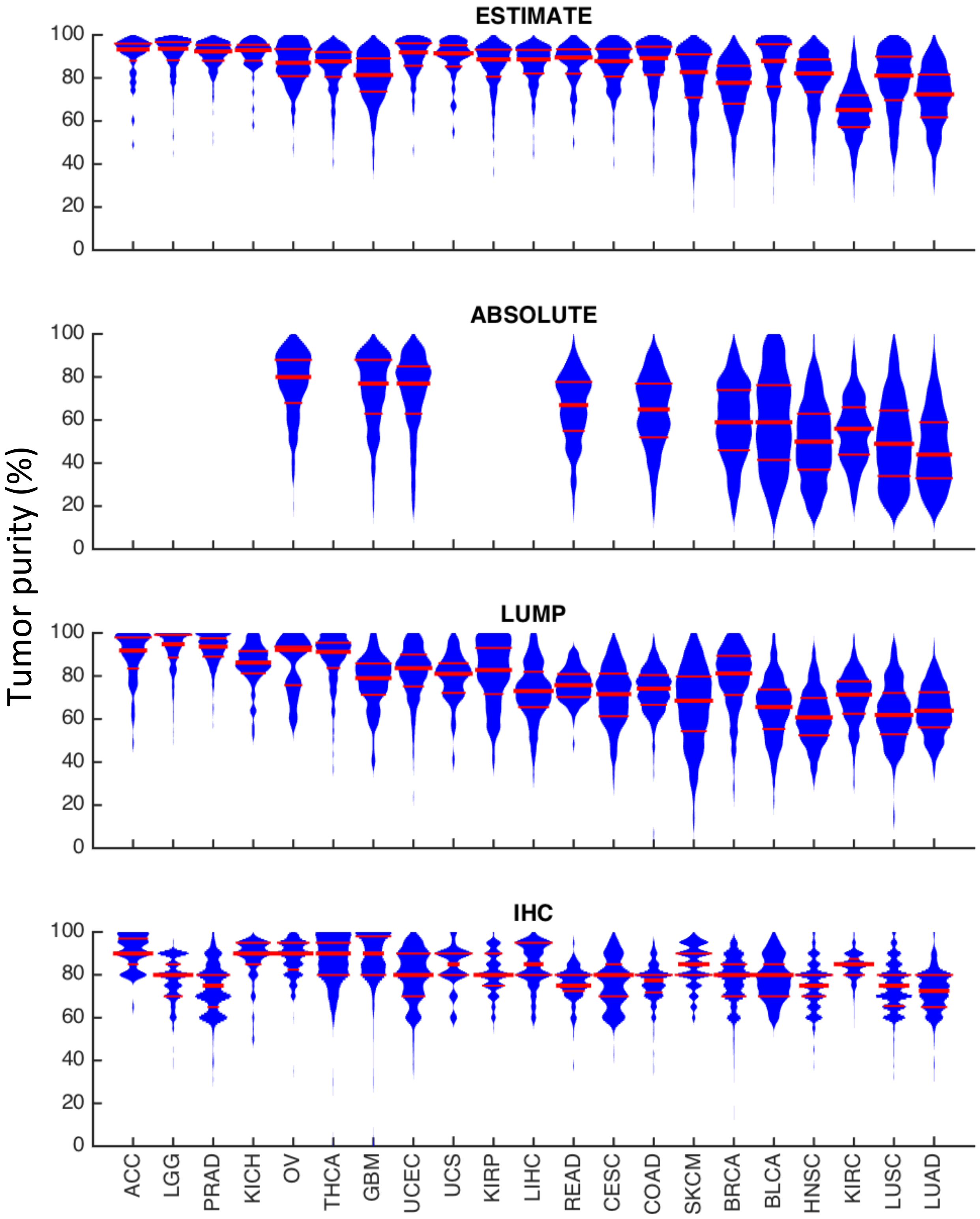

Tumor purity of TCGA cancers types. Violin plots of tumor purity in 21 cancer types of the four purity estimation methods. Strong red line represents the median, weak red lines are 25 and 75 percentiles. The cancers were ordered according to median purity of CPE.

Supplementary Figure 6. Tumor purity and mutational burden

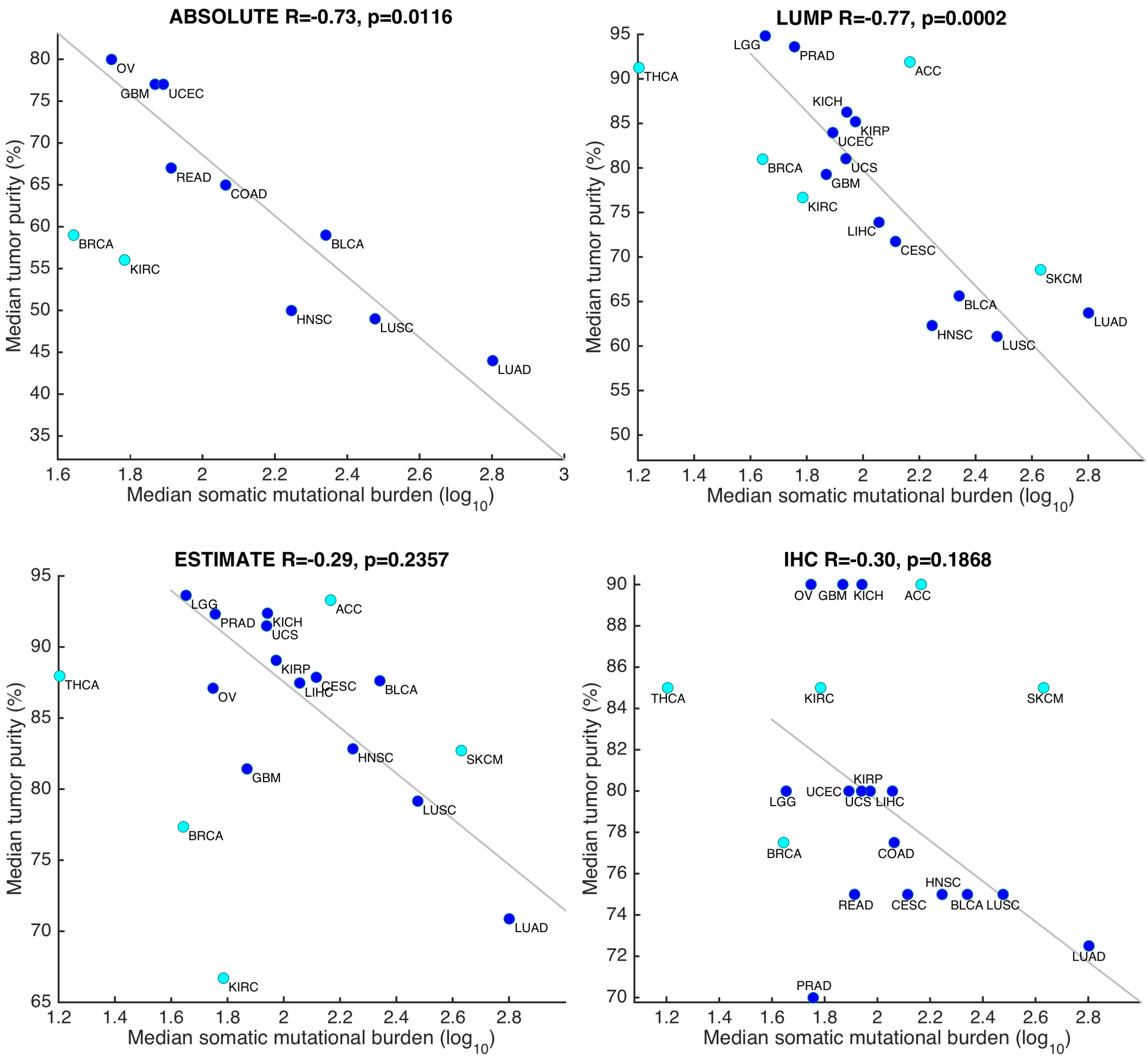

Tumor purity and mutational burden. Scatter plot of median number of mutations per tumor sample for each of the 21 cancer types (in log 10 scale) vs. the median tumor purity as calculated by the different methods. Pearson coefficient is presented. The least-squares line presented was calculated without the 5 outliers found with the CPE method (colored in cyan) to allow full comparison between the methods.

Supplementary Figure 7: Concordance between samples from the same patient.

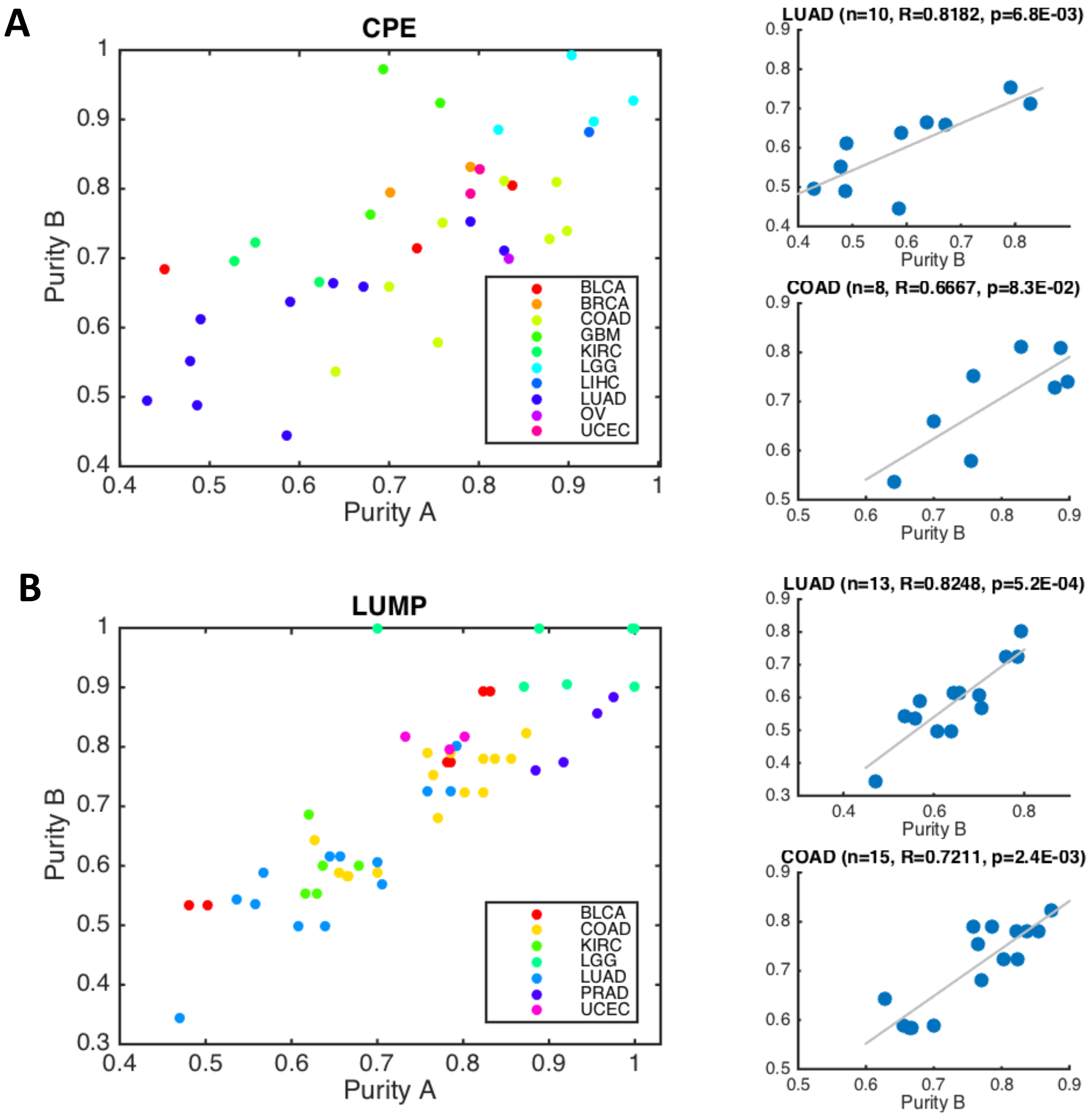

Concordance between samples from the same patient. A) 37 patients across cancer types with two distinct samples. The X axis shows purity measured by CPE of vial A, and the Y axis shows purity measured by CPE of vial B of the same patients. The Spearman coefficient for all patients is 0.73. It can be observed that the correlations are maintained within cancer types. The plots on the right show the same analysis for LUAD and COAD samples. **B)** DNA methylation profiling was performed 53 times for 2 samples from the same patients (much more than other methods). Thus, we performed the analysis again for patients with DNA methylation measurements and purity estimations are based on LUMP. The high correlation between and within samples is maintained. The plots on the right show the same analysis for LUAD and COAD samples.

* It should be noted that there are no patients that were analyzed twice with SNP array, therefore there is no ABSOLUTE data available for such analysis.

Supplementary Figure 8: Tumor purity and clinical features

A LGG histological types

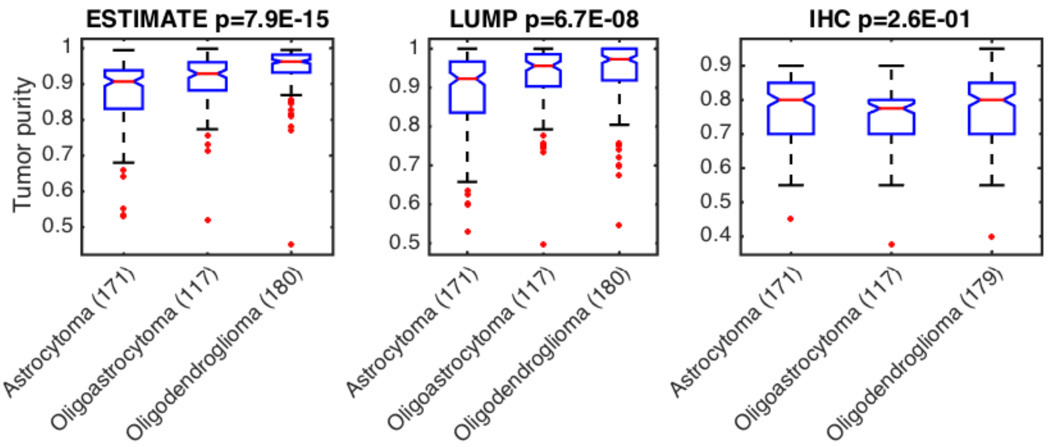

B CESC histological types

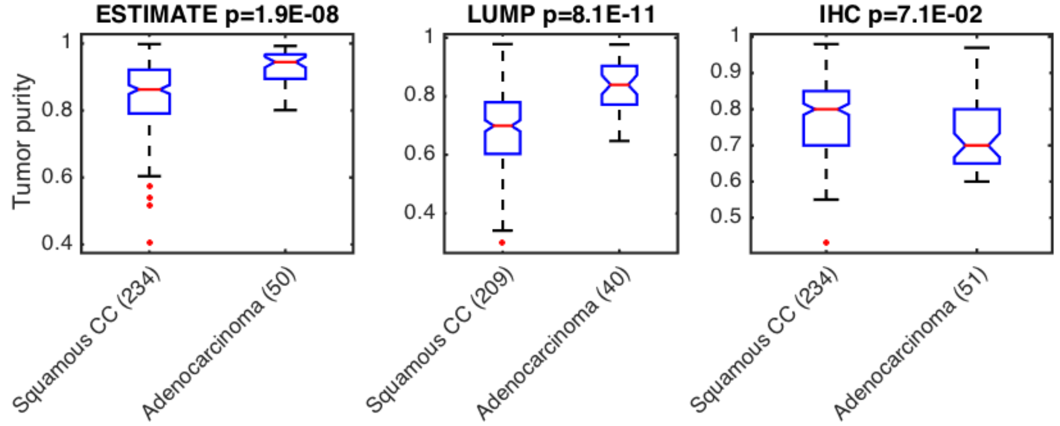

C BRCA histological types

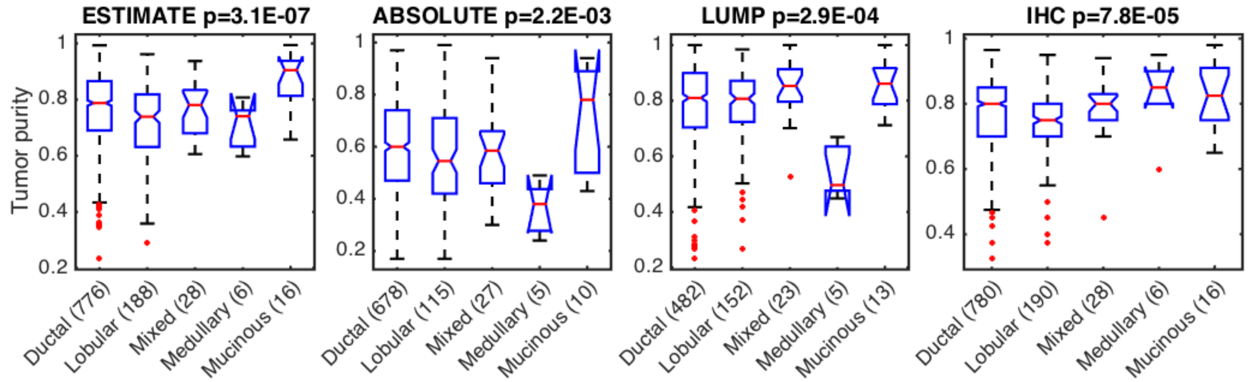

D **THCA histological types**

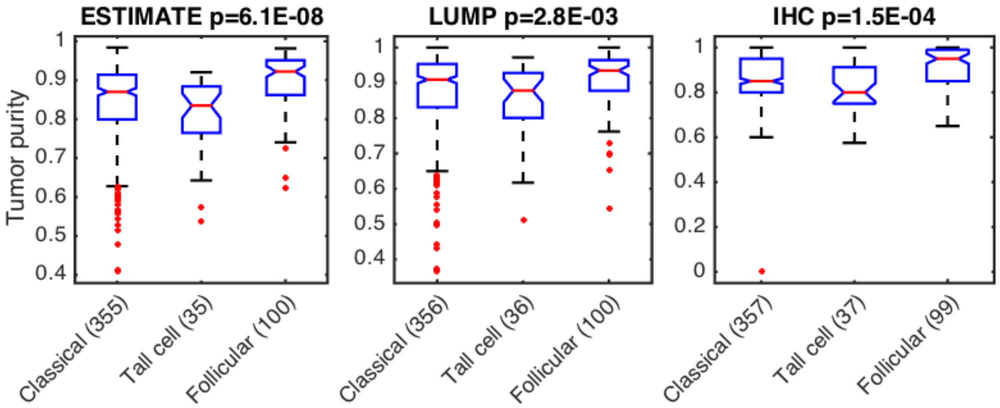

E **KIRC histological grades**

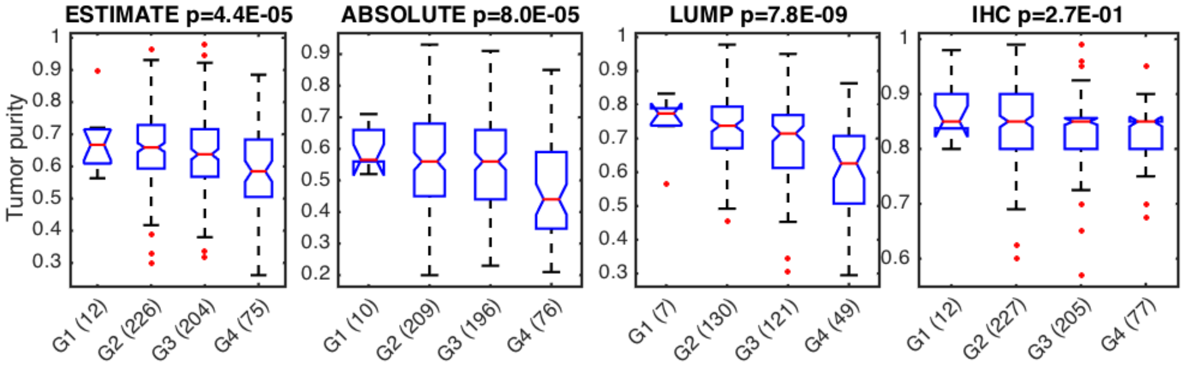

F **LGG histological grades**

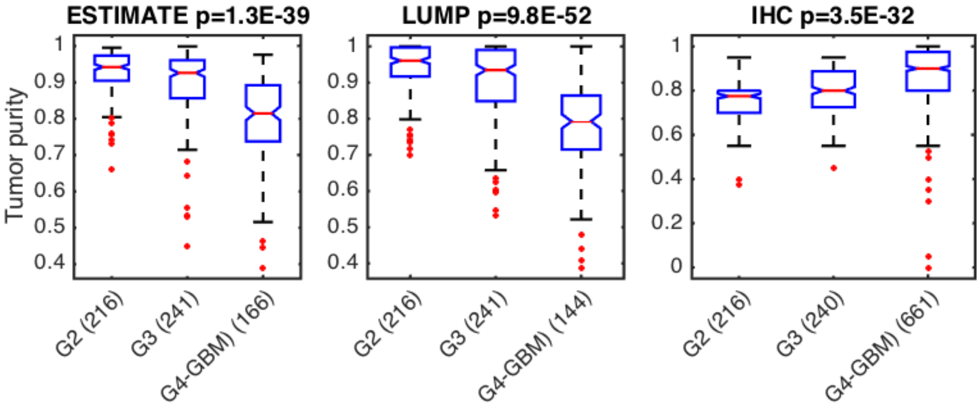

G **PRAD histological grades (primary)**

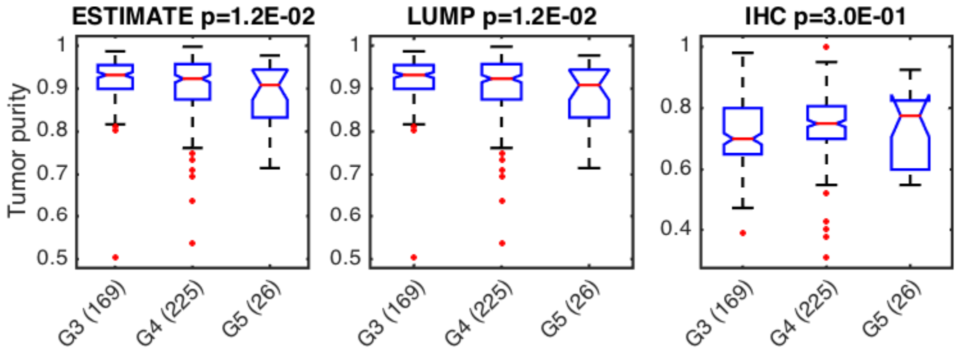

H LGG Tumor location

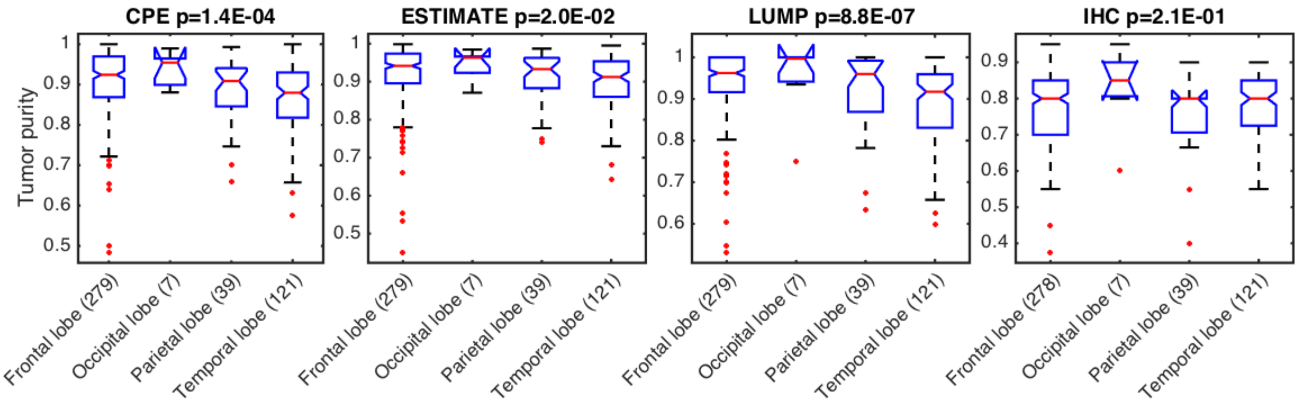

I BRCA estrogen receptor (ER)-status

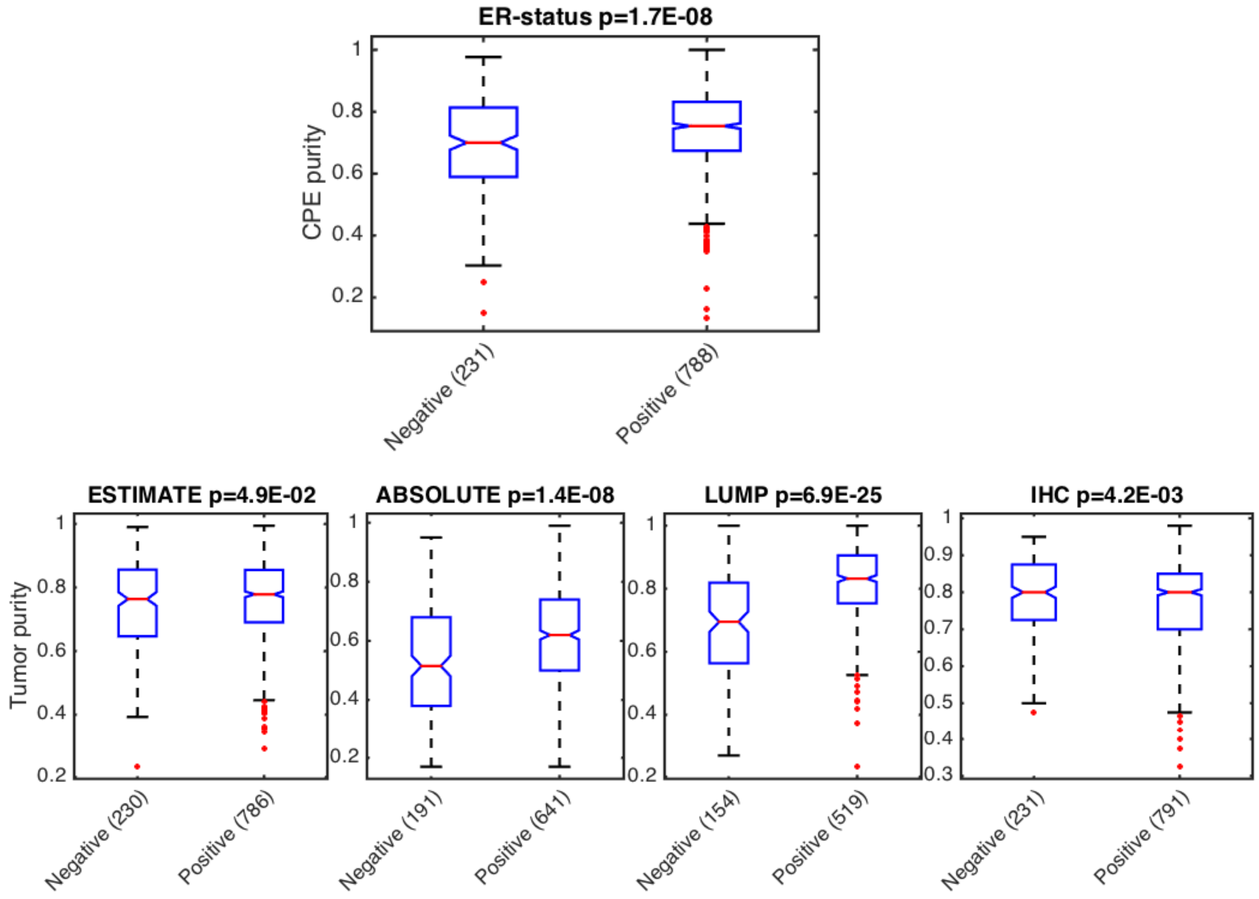

J LGG IDH1 mutation

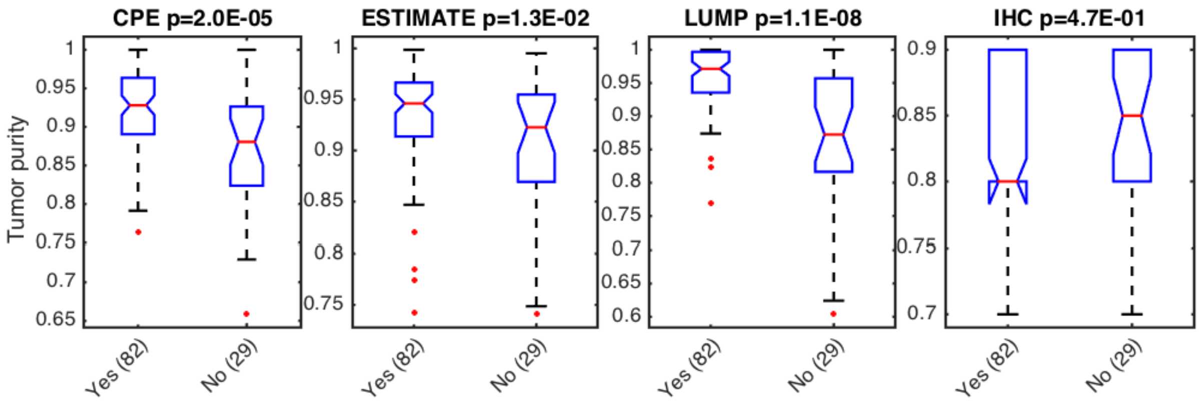

K THCA Patient's history of thyroid gland disorder

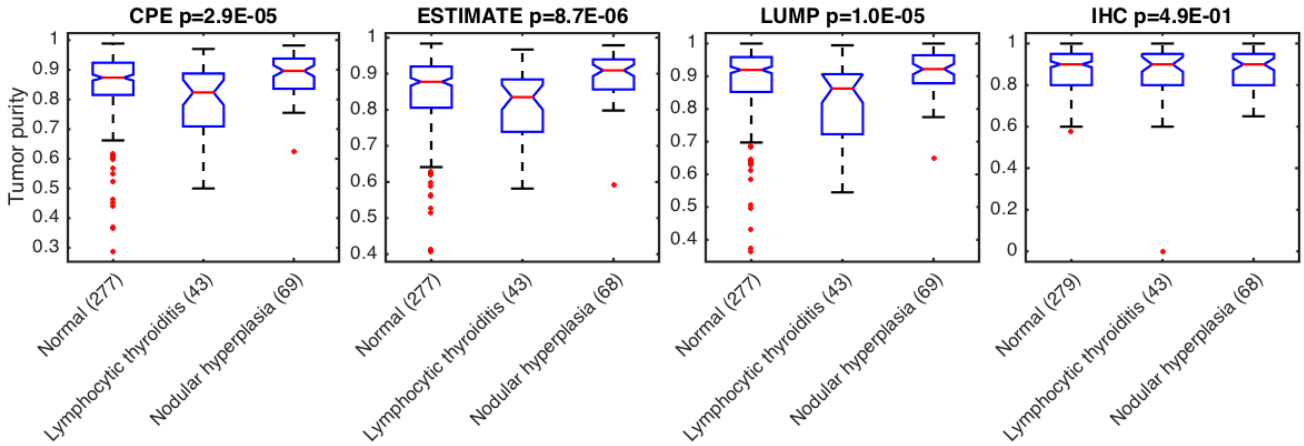

Tumor purity and clinical features. A-D) Box plots of the additional available purity methods for the histological subtypes presented in figure 3a. Central red mark is the median, the edges of the box are the 25th and 75th percentiles. **E-G)** Box plots of the additional available purity methods for the histological grades presented in figure 3b. **H-K)** Box plots of the available purity methods for all other significant clinical features (FDR<1%) not presented in figure 3.

Supplementary Figure 9: Co-expression and tumor purity

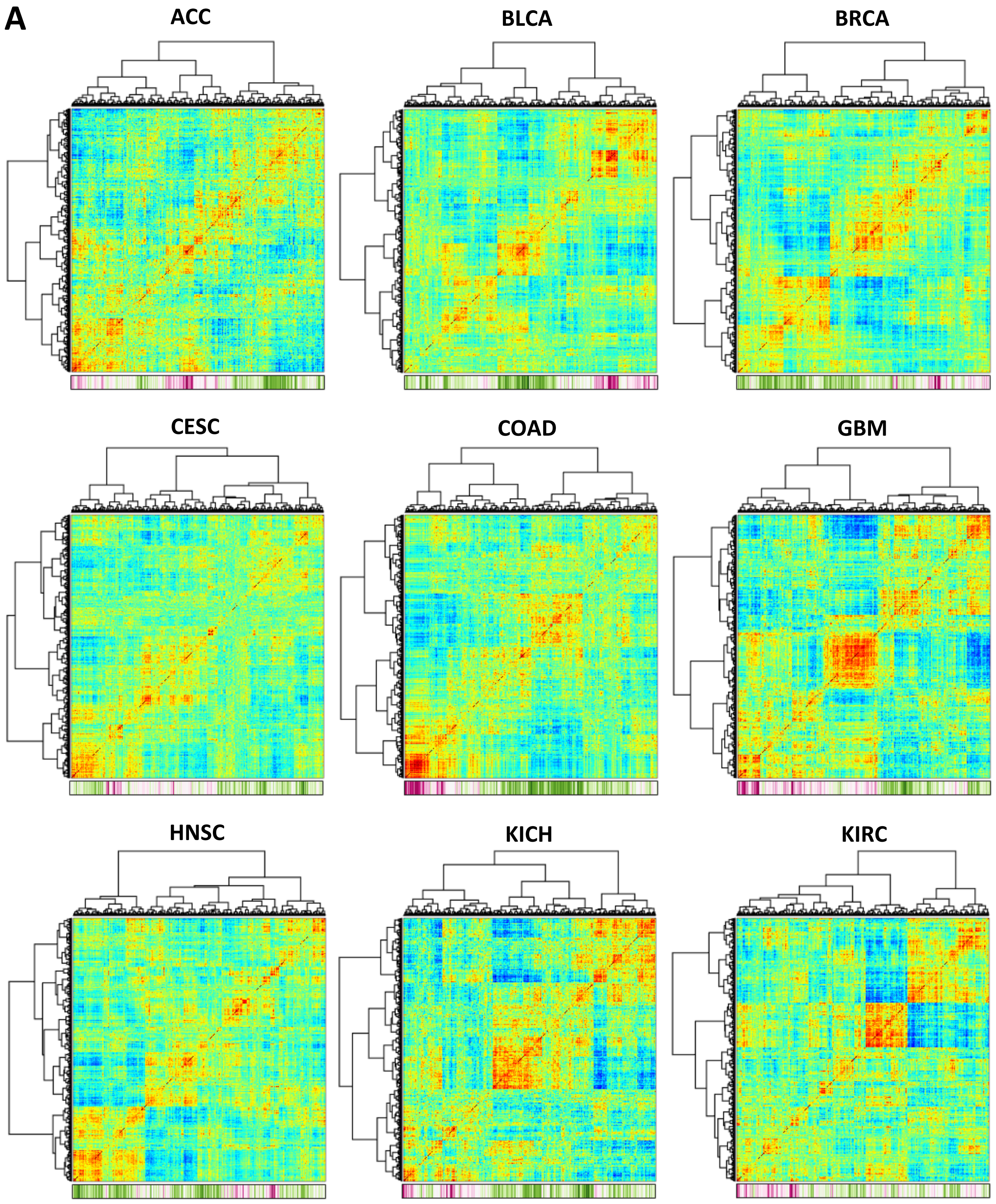

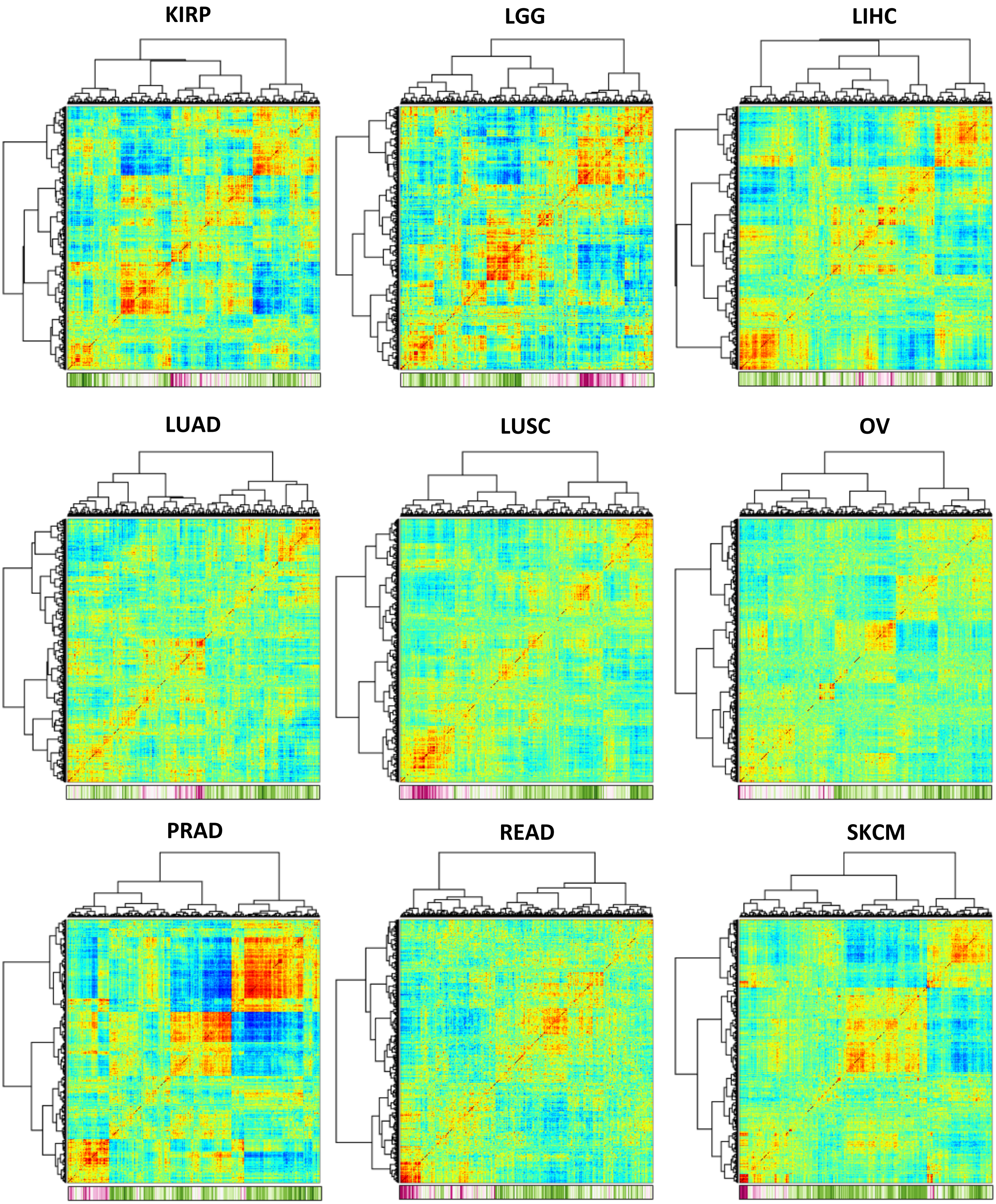
KIRP
LGG
LIHC
LUAD
LUSC
OV
PRAD
READ
SKCM

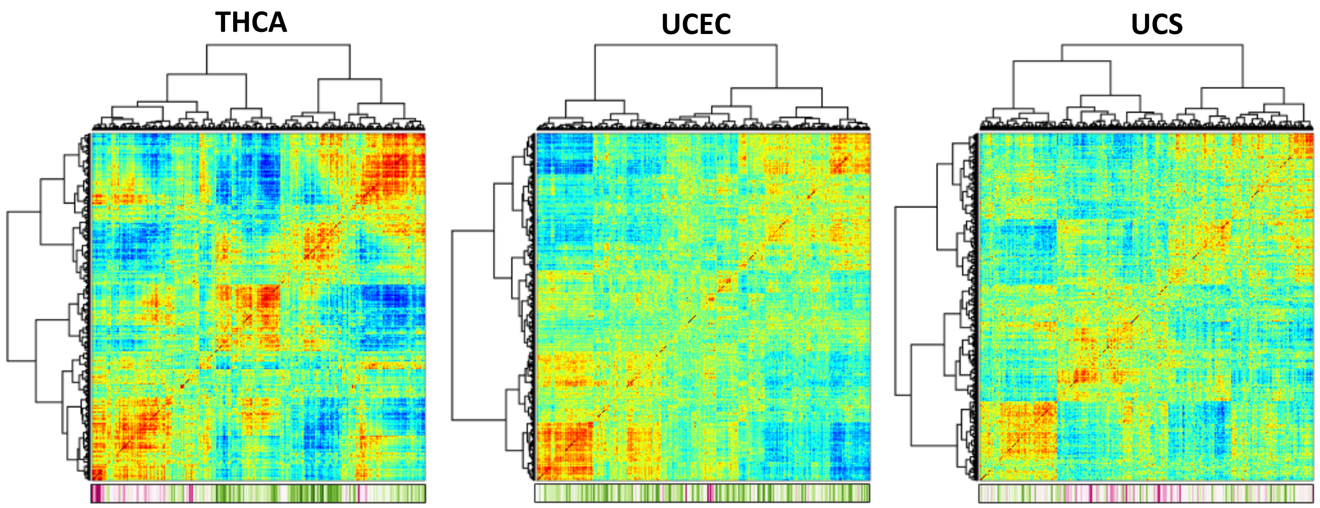

B

Cancer type	# of co-expressions (|r|>0.5)	# of gene pairs both correlated with purity (|r|>0.3)	# of co-expression & purity-associated pairs	# of pairs expected by random	% of coexpression and purity-asscoiated pairs	Fold-ratio
**ACC**	178,501	993,345	56,598	808.4	31.7	70.0
**BLCA**	155,744	419,986	77,454	965.2	49.7	80.2
**BRCA**	170,639	224,785	27,558	376.3	16.2	73.2
**CESC**	92,658	38,781	5,766	42.8	6.2	134.9
**COAD**	169,527	419,070	86,416	1,172.2	51.0	73.7
**GBM**	503,157	1,092,981	112,023	4,510.1	22.3	24.8
**HNSC**	171,524	87,571	13,881	190.5	8.1	72.9
**KICH**	498,317	943,251	78,044	3,111.9	15.7	25.1
**KIRC**	389,235	255,970	24,593	766.0	6.3	32.1
**KIRP**	411,132	216,811	20,852	686.0	5.1	30.4
**LGG**	526,346	1,836,486	241,149	10,156.3	45.8	23.7
**LIHC**	208,853	57,630	14,377	240.3	6.9	59.8
**LUAD**	74,341	245,350	20,543	122.2	27.6	168.1
**LUSC**	84,772	536,130	46,362	314.5	54.7	147.4
**OV**	40,465	103,740	7,822	25.3	19.3	308.8
**PRAD**	929,065	201,930	51,504	3,828.8	5.5	13.5
**READ**	157,432	365,085	56,160	707.5	35.7	79.4
**SKCM**	126,907	261,726	34,015	345.4	26.8	98.5
**THCA**	883,565	561,270	106,871	7,555.7	12.1	14.1
**UCEC**	254,209	35,245	4,690	95.4	1.8	49.2
**UCS**	195,673	21,945	1,609	25.2	0.8	63.9

Co-expression and tumor purity. A) Co-expression matrices of top 5000 differentiating genes in 21 cancer types as in figure 3b. **B)** Table shows the enrichment genes correlated with purity in co-expressing pairs.

Supplementary Figure 10: Co-expression analysis controlled by tumor purity

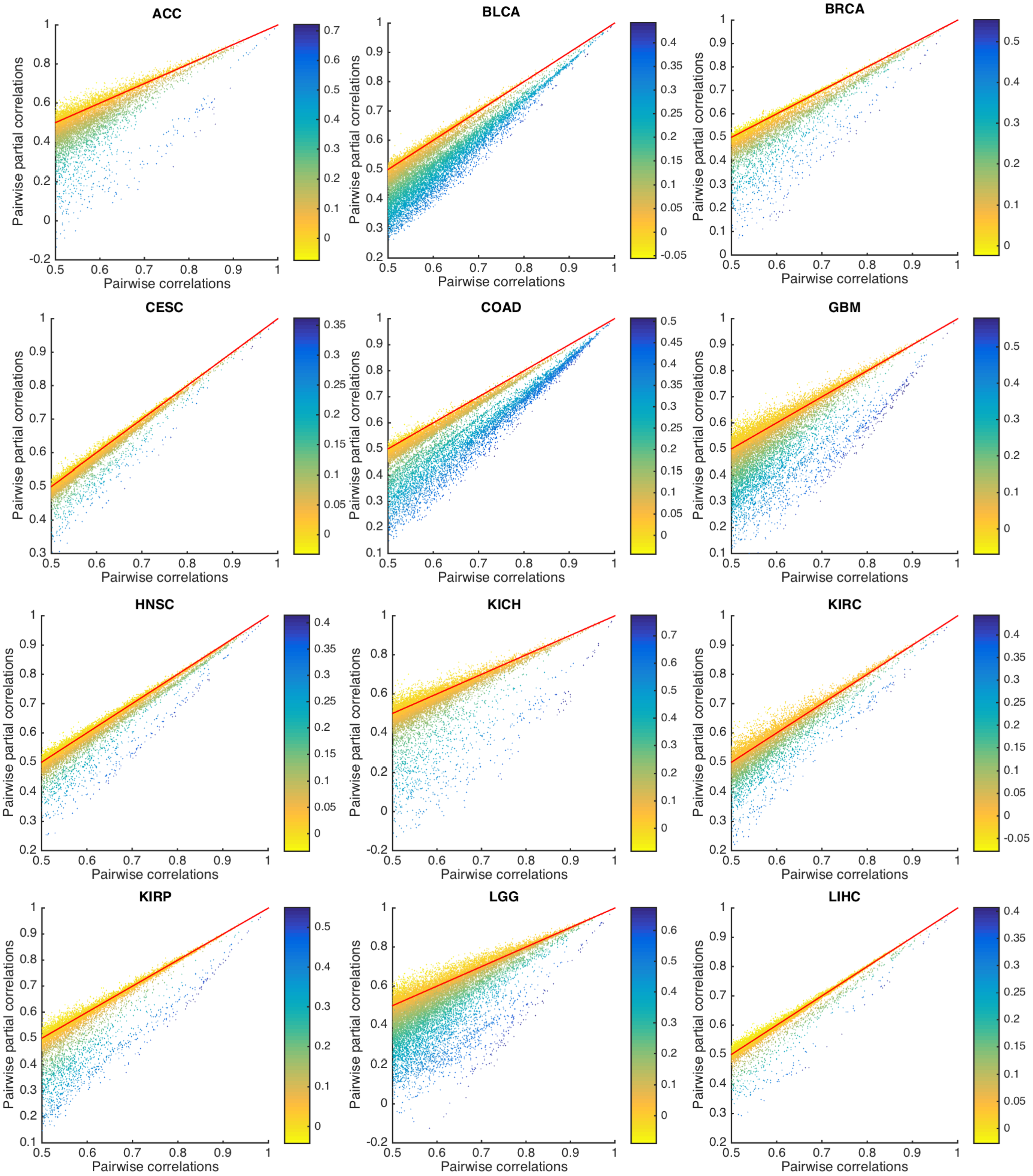

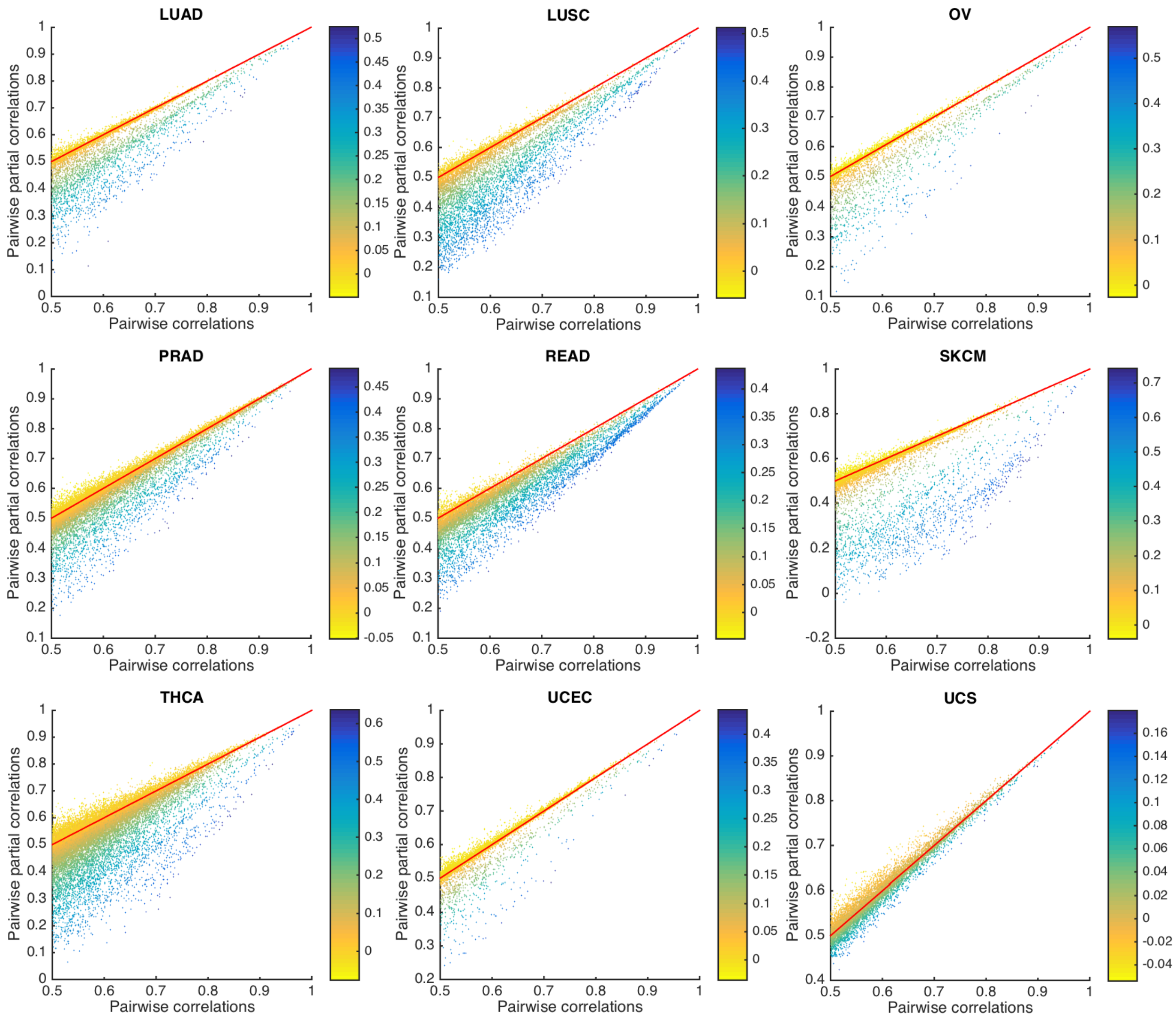

Co-expression analysis controlled by tumor purity. Scatter plots of co-expression correlations (x-axis) vs. partial correlation of co-expression controlling for CPE purity levels (y-axis) in each of the 21 cancer types. The analysis was restricted for top 1000 genes according to gene expression standard deviation in each cancer type, and the plot presents only correlation with a Spearman coefficient > 0.5. The colors correspond to the multiplication of the correlation of the co-expressed genes with purity.

Supplementary Figure 11: Tumor purity and molecular subtyping of GBM

A GBM Molecular subtyping

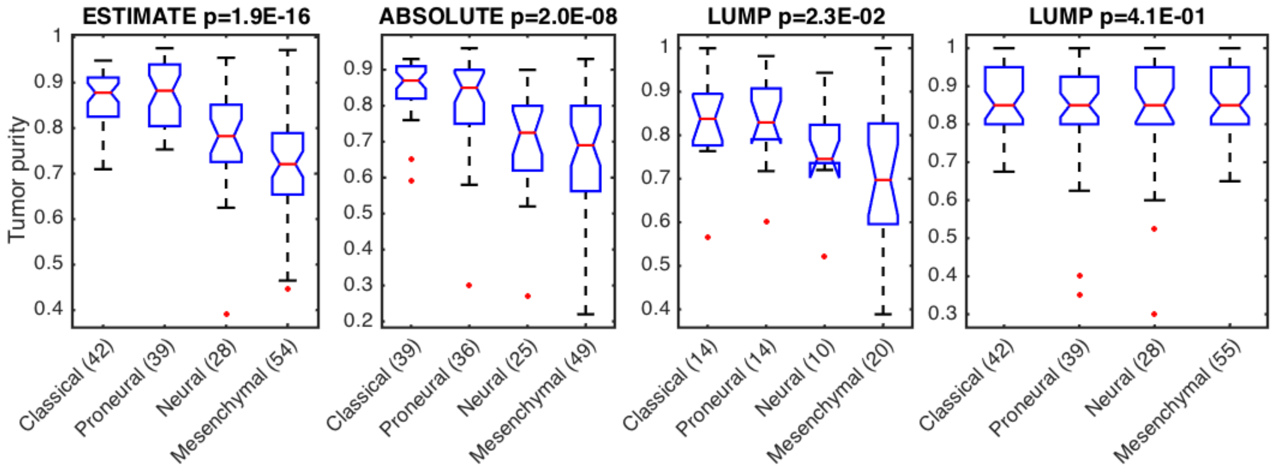

B GBM Molecular subtyping using U133A data

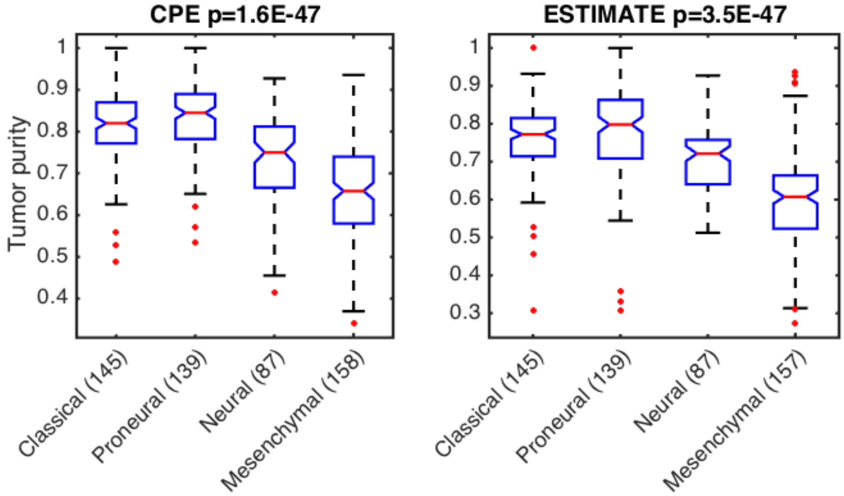

Tumor purity and molecular subtyping of GBM. A) Box plots of the additional available purity methods for the molecular subtyping of GBM. Central red mark is the median, the edges of the box are the 25th and 75th percentiles. **B)** TCGA contains 538 GBM samples analyzed for gene expression using the Affymetrix Human Genome U133A array. We calculate ESTIMATE purity levels for these samples, and accordingly the CPE levels. The results obtained using this data repeat the findings obtained using the RNA-seq data.

Supplementary Figure 12: Tumor purity and molecular subtyping of LUAD

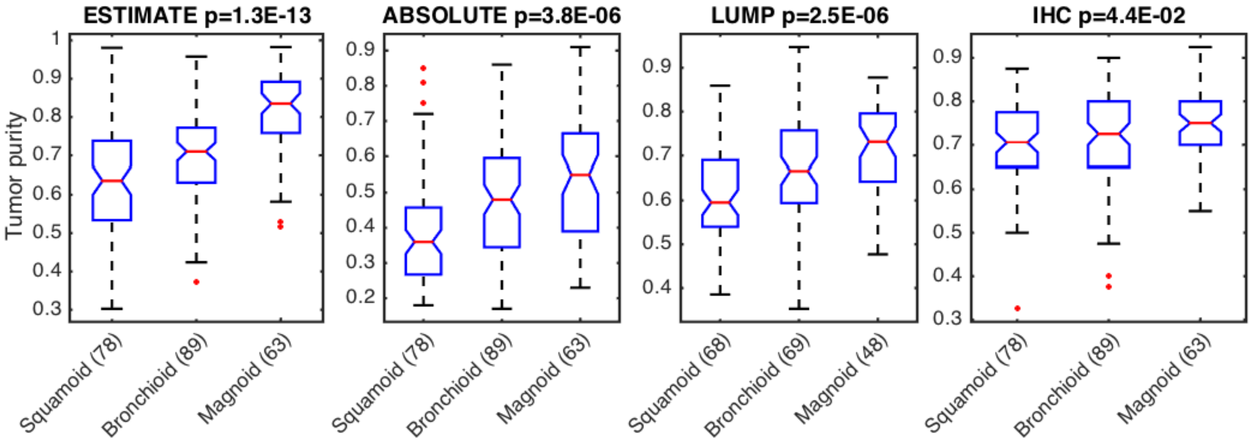

Tumor purity and molecular subtyping of LUAD. Box plots of the additional available purity methods for the molecular subtyping of LUAD. Central red mark is the median, the edges of the box are the 25th and 75th percentiles.

Supplementary Figure 13: Tumor purity and molecular subtyping of BRCA

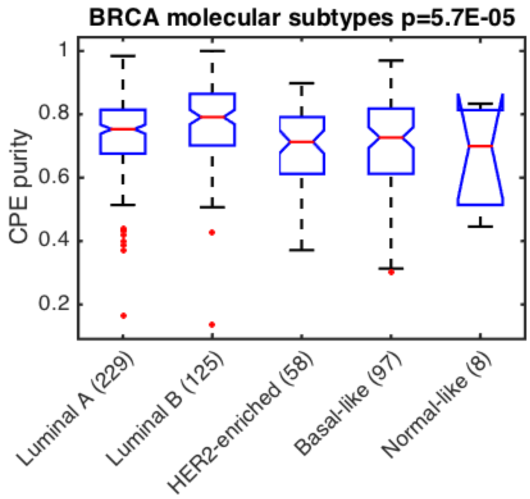

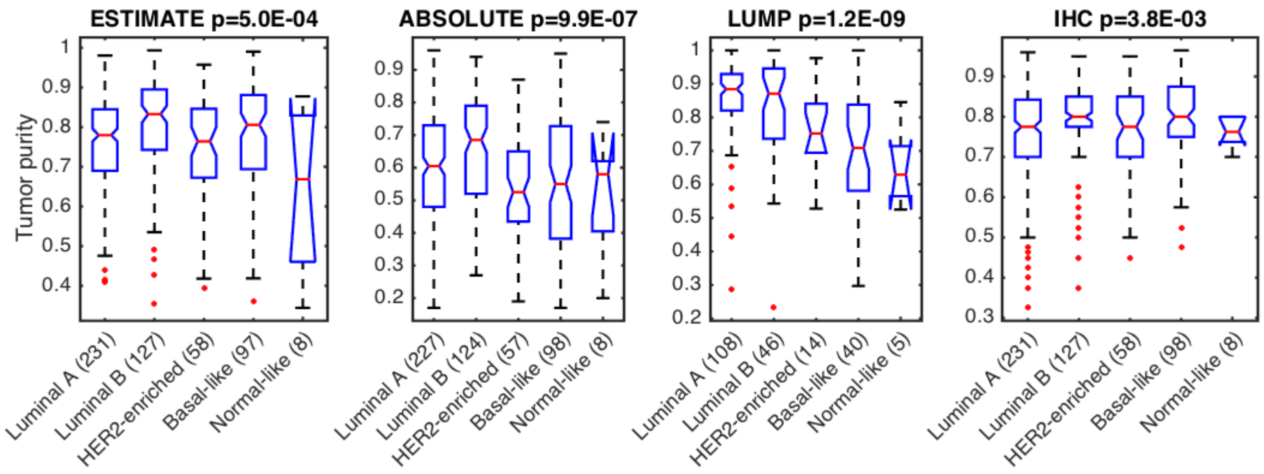

Tumor purity and molecular subtyping of BRCA. Box plots of breast cancer molecular subtyping by PAM50 as a function of purity. Luminal A/B show significantly higher purity levels, according to CPE, Estimate, ABSOLUTE and LUMP, than the other subtypes. One-way ANOVA p-value is presented above. Number of samples for each subtype is in parenthesis.

Supplementary Figure 14: Purity levels in non-tumor adjacent normal samples

A

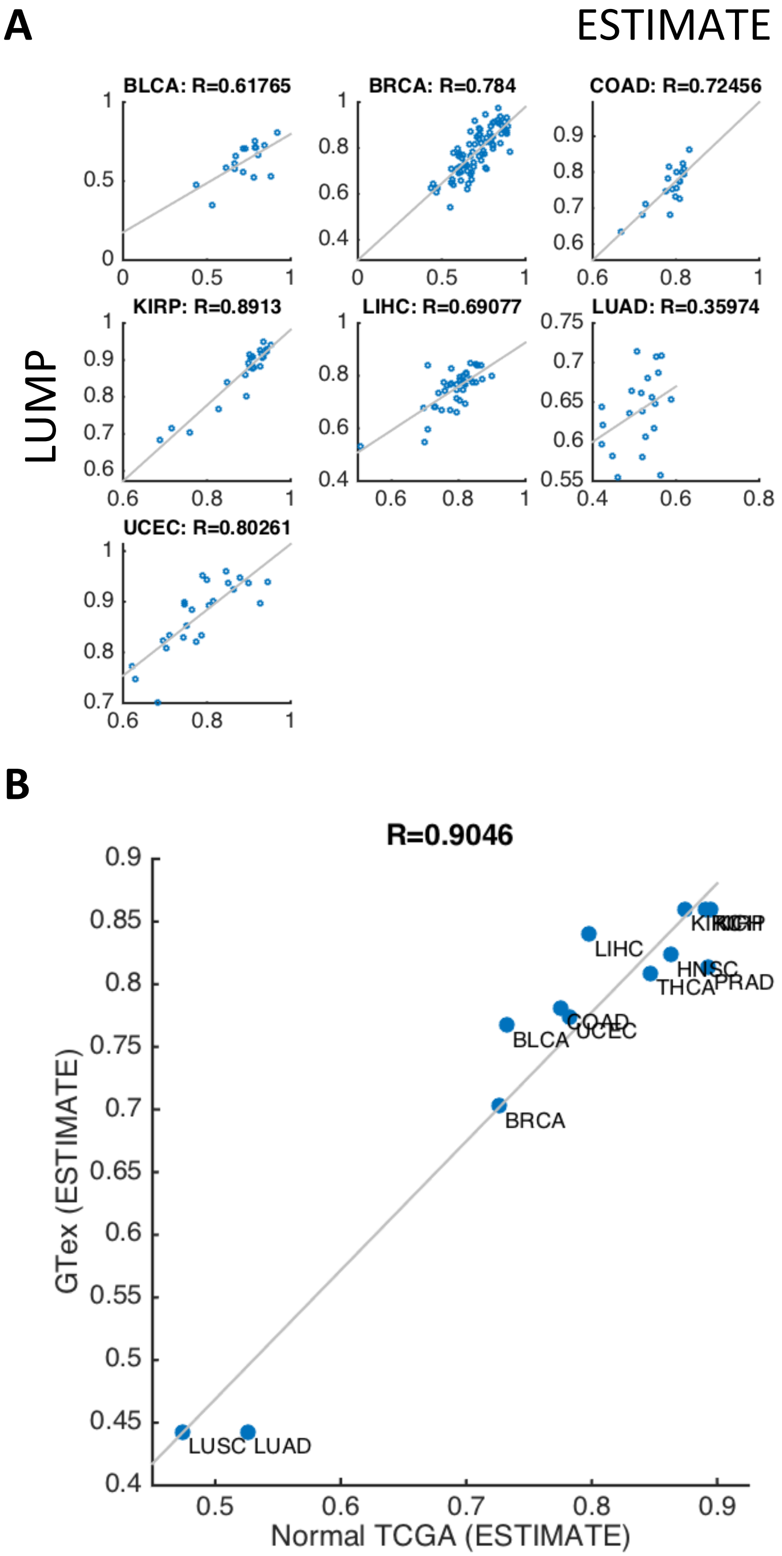

C

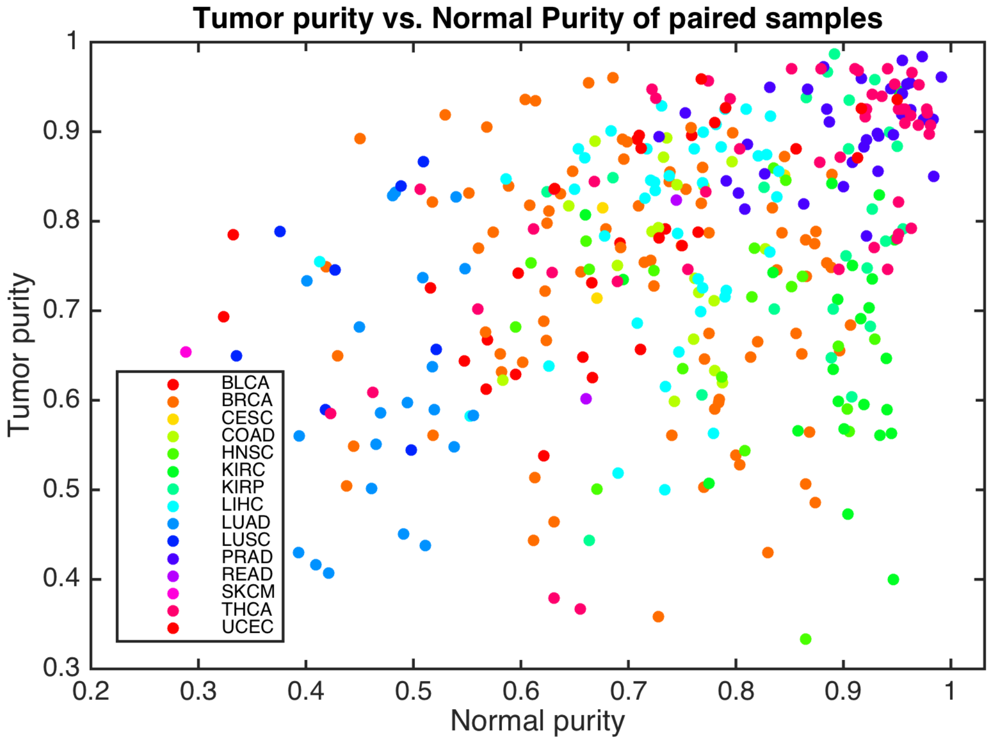
Tumor purity vs. Normal Purity of paired samples
Tumor purity
Normal purity
BLCA
BRCA
CESC
COAD
HNSC
KIRC
KIRP
LIHC
LUAD
LUSC
PRAD
READ
SKCM
THCA
UCEC
0.3
0.4
0.5
0.6
0.7
0.8
0.9
1
0.2

D

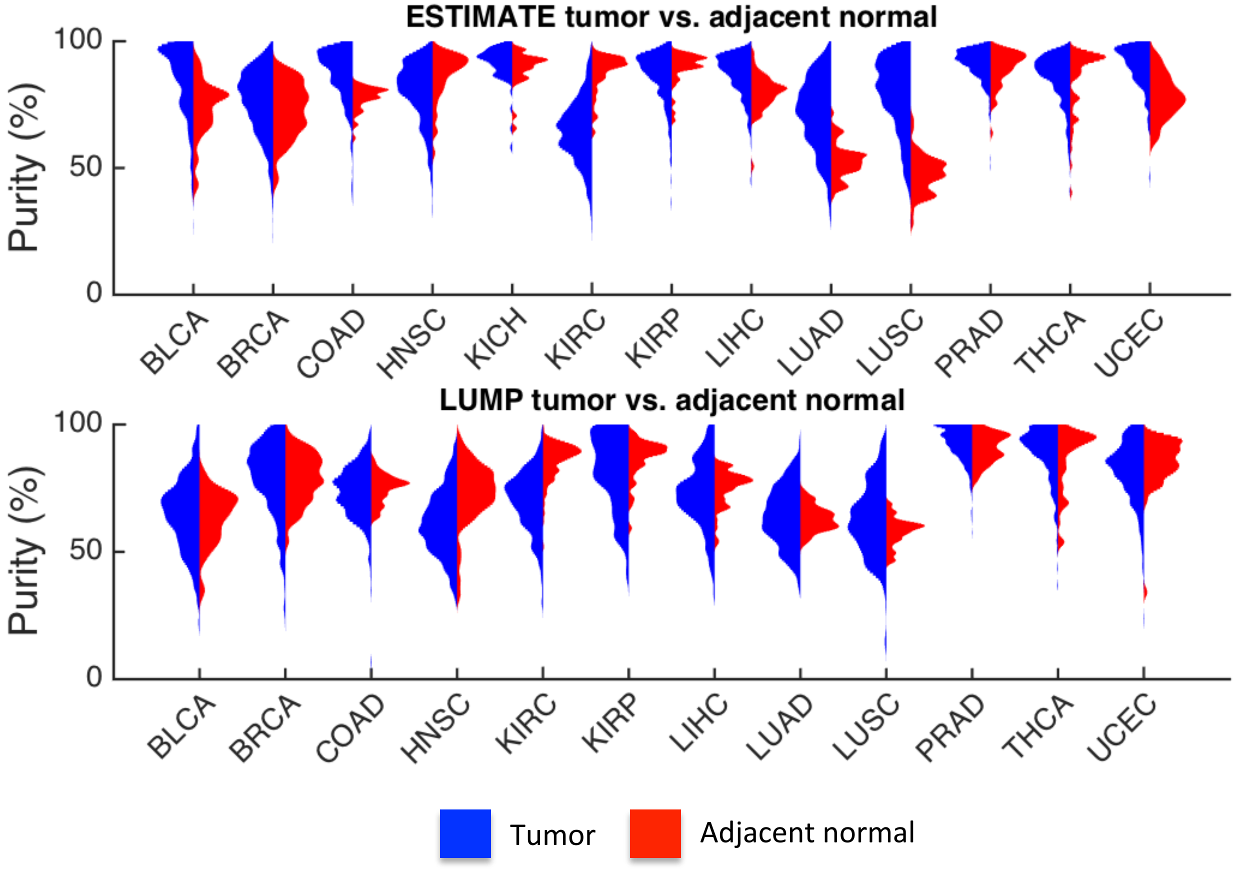
ESTIMATE tumor vs. adjacent normal
Purity (%)
100
50
0
BLCA
BRCA
COAD
HNSC
KICH
KIRC
KIRP
LIHC
LUAD
LUSC
PRAD
THCA
UCEC
LUMP tumor vs. adjacent normal
Purity (%)
100
50
0
BLCA
BRCA
COAD
HNSC
KIRC
KIRP
LIHC
LUAD
LUSC
PRAD
THCA
UCEC
Tumor
Adjacent normal

Purity levels in non-tumor adjacent normal samples. A) Correlation between ESTIMATE and LUMP purity estimations for adjacent normal (normal) samples from 11 cancer types with sufficient information from both methods. We observe high correlations in 10 of the cancer types, and relatively lower correlation in normal samples of lung adenocarcinoma (LUAD). However, the variation in purity estimations of normal LUAD samples is low in both methods. B) Average ESTIMATE purity levels of normal samples from 12 TCGA cancer types (x-axis) vs. average ESTIMATE purity levels of samples from corresponding tissues taken from the Genotype-Tissue Expression project (GTEx). For example, the BLCA point the average purity from 17 normal samples adjacent to bladder carcinoma samples from TCGA and 11 bladder samples from GTEx. **C)** Purity levels in matched pairs. Y-axis is the CPE purity of the tumor samples and the x-axis is the purity of the adjacent normal sample of the same patient. In many cancer types there is no similarity between the two measurements. **D)** Violin plots of ESTIMATE (top) and LUMP (bottom) purity levels in 13 TCGA types. Blue distributions are for the tumor samples and red distributions are for the normal samples.

Supplementary Figure 15: Comparison of pre and post-adjustment to purity differentially expression experiments

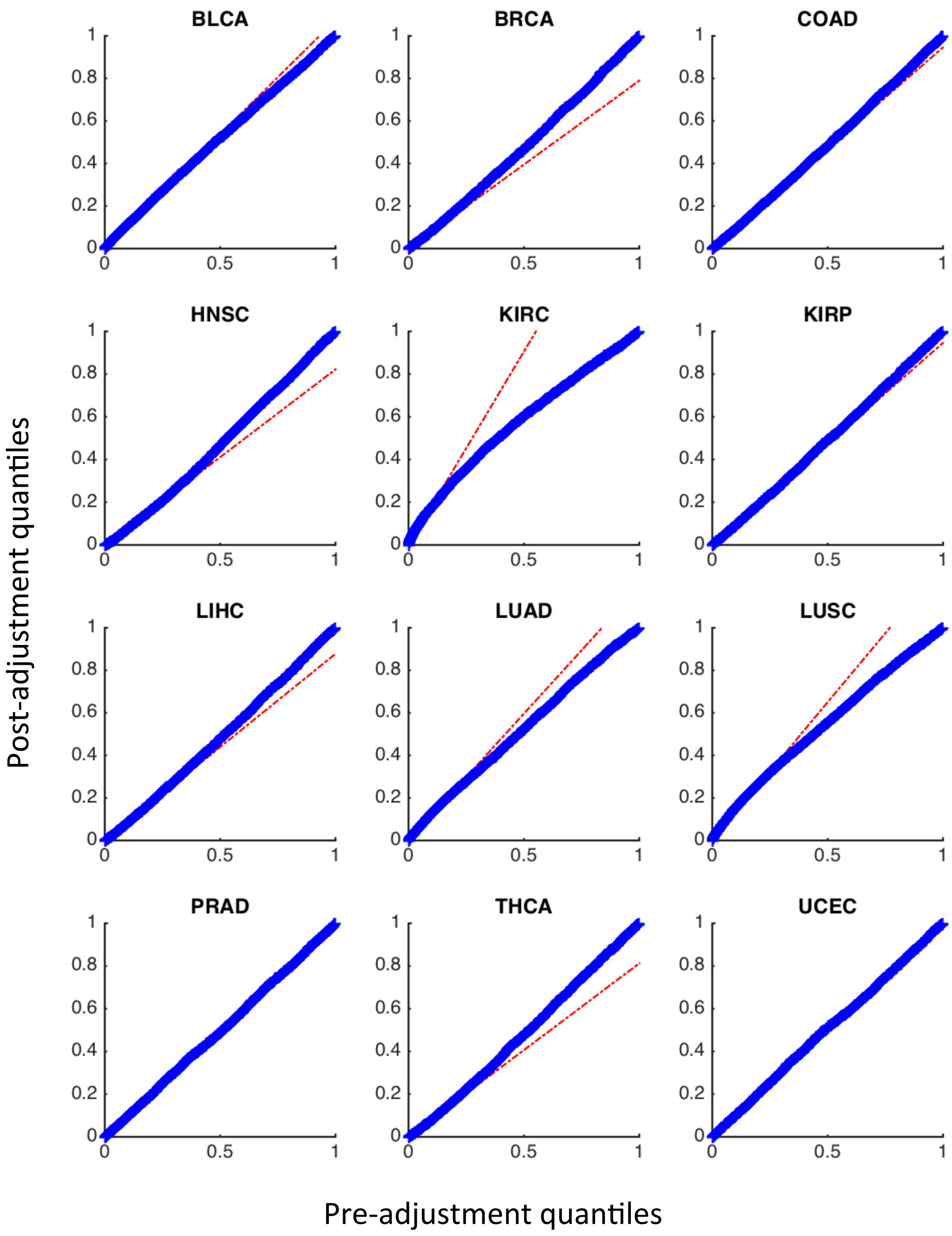

Comparison of pre and post-adjustment to purity differentially expression experiments. Q-Q plots for each cancer type, showing the p-value changes before and after the adjustment of the differential expression experiments.

Supplementary Table 1: TCGA samples analyzed in manuscript.

TCGA code	Cancer type	ESTIMATE (Gene expression)		ABSOLUTE (CNV)		LUMP (DNA methylation)		IHC (Immuno-histochemistry)		CPE (consensus)	
		Tumor	Normal	Tumor	Normal	Tumor	Normal	Tumor	Normal	Tumor	Normal
ACC	Adrenocortical carcinoma	79	0	0	0	80	0	92	4	80	0
BLCA	Bladder Urothelial Carcinoma	407	19	138	0	373	21	416	37	411	17
BRCA	Breast invasive carcinoma	1098	111	880	0	737	97	1120	163	1096	82
CESC	Cervical squamous cell carcinoma and endocervical adenocarcinoma	306	3	0	0	257	3	310	8	305	3
COAD	Colon adenocarcinoma	287	41	422	0	301	38	474	93	469	19
GBM	Glioblastoma multiforme	166	0	580	0	142	2	628	33	605	0
HNSC	Head and Neck squamous cell carcinoma	516	43	310	0	530	50	530	78	528	20
KICH	Kidney Chromophobe	66	25	0	0	66	0	113	71	66	0
KIRC	Kidney renal clear cell carcinoma	534	72	497	0	325	160	542	442	539	24
KIRP	Kidney renal papillary cell carcinoma	291	32	0	0	226	45	292	88	291	23
LGG	Brain Lower Grade Glioma	530	0	0	0	534	0	533	0	520	0
LIHC	Liver hepatocellular carcinoma	373	50	0	0	292	50	380	89	375	41
LUAD	Lung adenocarcinoma	513	58	357	0	466	32	537	211	530	21
LUSC	Lung squamous cell carcinoma	501	51	344	0	359	42	511	242	504	8
OV	Ovarian serous cystadenocarcinoma	265	0	567	0	10	0	608	97	581	0
PRAD	Prostate adenocarcinoma	498	52	0	0	429	50	499	118	498	35
READ	Rectum adenocarcinoma	95	9	164	0	99	7	173	19	167	2
SKCM	Skin Cutaneous Melanoma	471	1	0	0	461	2	474	3	470	1
THCA	Thyroid carcinoma	509	59	0	0	511	56	514	97	503	50
UCEC	Uterine Corpus Endometrial Carcinoma	175	24	498	0	439	46	553	52	551	24
UCS	Uterine Carcinosarcoma	57	0	0	0	57	0	57	6	57	0

TCGA samples analyzed in manuscript. Number of tumor and adjacent normal cases in TCGA analyzed by each of the five methods.

Supplementary Table 2: Tumor purity and prognosis across cancer types.

	ESTIMATE	METH	ABSOLUTE	IHC	CPE
LGG	1.04E-03	2.36E-07	NaN	5.36E-01	1.92E-05
KIRC	1.39E-01	3.55E-05	7.71E-04	9.09E-01	3.24E-03
GBM	1.00E-01	4.66E-03	9.86E-02	9.70E-02	8.38E-03
SKCM	1.97E-02	9.36E-02	NaN	3.75E-01	1.32E-02
READ	4.09E-01	4.30E-02	1.66E-01	4.15E-01	4.18E-02
CESC	1.32E-01	1.08E-01	NaN	8.93E-01	1.64E-01
BRCA	1.45E-01	9.09E-01	3.38E-01	2.37E-01	1.95E-01
UCEC	7.80E-01	4.41E-01	1.70E-01	6.00E-01	2.38E-01
LUSC	1.83E-01	9.97E-01	1.56E-01	1.72E-01	2.51E-01
HNSC	1.33E-01	2.09E-01	5.61E-01	7.69E-01	3.42E-01
LUAD	8.05E-01	5.47E-01	2.54E-01	2.46E-01	5.03E-01
UCS	6.73E-01	6.22E-01	NaN	1.72E-01	5.47E-01
KICH	4.78E-01	9.43E-01	NaN	2.47E-01	5.53E-01
ACC	8.67E-01	1.59E-01	NaN	1.72E-02	5.89E-01
KIRP	3.02E-01	2.50E-01	NaN	5.06E-01	6.19E-01
COAD	9.48E-01	6.62E-01	9.44E-01	8.74E-01	7.16E-01
LIHC	7.37E-01	8.18E-01	NaN	3.40E-01	7.47E-01
OV	8.34E-01	2.68E-01	8.39E-01	9.14E-01	7.84E-01
BLCA	5.84E-01	7.14E-01	1.05E-01	5.76E-01	8.12E-01
THCA	3.50E-01	3.77E-01	NaN	2.73E-01	8.86E-01
PRAD	7.27E-01	9.50E-01	NaN	8.51E-01	9.94E-01

Tumor purity and prognosis across cancer types. P-values of Cox proportional hazard regression analysis of survival time with censoring for 21 cancer types in 5 purity estimation methods. Lower grade glioma (LGG) and kidney renal cell carcinoma (KIRC) are significant in three methods, and pass multiple hypothesis correction.

Supplementary Table 3: Enriched GO annotations of co-expressing genes associated with purity.

GO term	Description	P-value (Hypergeometric)	FDR q-value	Enrichment
**GO:0030198**	extracellular matrix organization	4.15E-45	4.32E-41	2.74
**GO:0043062**	extracellular structure organization	4.15E-45	2.16E-41	2.74
**GO:0007155**	cell adhesion	1.85E-35	6.41E-32	2.09
**GO:0022610**	biological adhesion	3.68E-35	9.60E-32	2.08
**GO:0002376**	immune system process	8.80E-30	1.83E-26	1.71
**GO:0006952**	defense response	3.64E-28	6.32E-25	1.87
**GO:0006955**	immune response	3.38E-23	5.02E-20	1.86
**GO:0006928**	movement of cell or subcellular component	8.62E-23	1.12E-19	1.71
**GO:0002682**	regulation of immune system process	1.24E-22	1.43E-19	1.77
**GO:0002684**	positive regulation of immune system process	7.18E-22	7.48E-19	1.97
**GO:0030334**	regulation of cell migration	9.23E-22	8.74E-19	2.03
**GO:0032501**	multicellular organismal process	2.54E-21	2.21E-18	1.46
**GO:0044707**	single-multicellular organism process	2.96E-21	2.37E-18	1.47
**GO:2000145**	regulation of cell motility	3.56E-21	2.65E-18	1.99
**GO:0048583**	regulation of response to stimulus	9.49E-21	6.59E-18	1.41
**GO:0050896**	response to stimulus	1.42E-20	9.25E-18	1.33
**GO:0051270**	regulation of cellular component movement	2.12E-20	1.30E-17	1.94
**GO:0051239**	regulation of multicellular organismal process	2.25E-20	1.30E-17	1.52
**GO:0007166**	cell surface receptor signaling pathway	3.19E-20	1.75E-17	1.51
**GO:0009605**	response to external stimulus	5.93E-20	3.09E-17	1.71
**GO:0040012**	regulation of locomotion	7.37E-20	3.66E-17	1.92
**GO:0048870**	cell motility	1.17E-19	5.56E-17	1.87
**GO:0022617**	extracellular matrix disassembly	2.13E-19	9.65E-17	2.85
**GO:0006954**	inflammatory response	3.44E-19	1.49E-16	2.35
**GO:0016477**	cell migration	5.52E-19	2.30E-16	1.87
**GO:0048584**	positive regulation of response to stimulus	5.54E-19	2.22E-16	1.54
**GO:0043207**	response to external biotic stimulus	1.97E-18	7.61E-16	1.93
**GO:0007165**	signal transduction	2.52E-18	9.37E-16	1.33
**GO:0009607**	response to biotic stimulus	7.99E-18	2.87E-15	1.89
**GO:0040011**	locomotion	1.11E-17	3.86E-15	1.75
**GO:0032963**	collagen metabolic process	1.51E-16	5.09E-14	3.04
**GO:0030335**	positive regulation of cell migration	1.87E-16	6.08E-14	2.2
**GO:0006950**	response to stress	2.90E-16	9.14E-14	1.38
**GO:0019221**	cytokine-mediated signaling pathway	4.54E-16	1.39E-13	2.1
**GO:0030155**	regulation of cell adhesion	5.16E-16	1.53E-13	1.9
**GO:0050776**	regulation of immune response	8.65E-16	2.50E-13	1.79
**GO:0050778**	positive regulation of immune response	8.92E-16	2.51E-13	2.01
**GO:2000147**	positive regulation of cell motility	9.57E-16	2.62E-13	2.16

Enriched GO annotations of co-expressing genes associated with purity. Top GO annotations that were enriched for the group of genes A in figure 3b. These genes have a high expression correlation between them, but also tend to be highly correlated with tumor purity. The table shows 'high-level' annotations of the immune system, but also for many other molecular pathways.
